# Education of staff in preschool aged classrooms in child care centers and child outcomes: A meta-analysis and systematic review

**DOI:** 10.1371/journal.pone.0183673

**Published:** 2017-08-30

**Authors:** Olesya Falenchuk, Michal Perlman, Evelyn McMullen, Brooke Fletcher, Prakesh S. Shah

**Affiliations:** 1 Applied Psychology and Human Development, University of Toronto/OISE, Toronto, Ontario, Canada; 2 Department of Gastroenterology, Alberta Children's Hospital, Calgary, Alberta, Canada; 3 Department of Pediatrics, Mount Sinai Hospital, Toronto, Ontario, Canada; 4 Department of Pediatrics, University of Toronto, Toronto, Ontario, Canada; 5 Institute of Health Policy, Management and Evaluation, University of Toronto, Toronto, Ontario, Canada; Kyoto University, JAPAN

## Abstract

Staff education is considered key to quality of early childhood education and care (ECEC) programs. However, findings about associations between staff education and children’s outcomes have been inconsistent. We conducted a systematic review and meta-analysis of associations between ECEC staff education and child outcomes. Searches of Medline, PsycINFO, and ERIC, websites of large datasets and reference sections of all retrieved articles were conducted. Eligible studies provided a statistical link between staff education and child outcomes for preschool-aged children in ECEC programs. Titles, abstracts and paper reviews as well as all data extraction were conducted by two independent raters. Of the 823 studies reviewed for eligibility, 39 met our inclusion criteria. Research in this area is observational in nature and subject to the inherent biases of that research design. Results from our systematic review were hampered by heterogeneity in how staff education was defined, variability in whose education was measured and the child outcomes that were assessed. However, overall the qualitative summary indicates that associations between staff education and childhood outcomes are non-existent to very borderline positive. In our meta-analysis of more homogeneous studies we identified certain positive, albeit very weak, associations between staff education and children’s language outcomes (specifically, vocabulary and letter word identification) and no significant association with a mathematics outcome (WJ Applied Problems). Thus, our findings suggest that within the range of education levels found in the existing literature, education is not a key driver of child outcomes. However, since we only explored levels of education that were reported in the literature, our findings cannot be used to argue for lowering education standards in ECEC settings.

## Introduction

Over 60% of children under age six regularly attend some type of out-of-home child care program in the USA [1–3]]. Exposure to early childhood education and care (ECEC) is thought to positively influence children’s pre-academic skills including improvements in cognitive, language and social/emotional abilities [4] Furthermore, exposure to ECEC programs can reduce gaps in academic performance caused by social inequalities [5], as exposure to high quality environments has been shown to be most beneficial for children who come from at-risk backgrounds. However, research findings suggest that these benefits are only realized when quality of care is good [6]. For example, children exposed to better quality ECEC programs scored higher on measures of numeracy, receptive vocabulary and school readiness when compared to children who had been exposed to lower quality care [7]. Others have shown links between the quality of care and positive social outcomes for children [8,9]. Unfortunately, research on quality of care suggests that child care quality is generally mediocre at best [10,11]. High ECEC utilization rates and the frequently inadequate quality of programs have raised questions about which aspects of ECEC programs are important to child outcomes.

Quality in the ECEC context is comprised of structural and process characteristics. Structural quality characteristics include constructs such as staff/child ratios, group size and staff education. [12] These are generally quantifiable and therefore easier to regulate by government. Structural quality is thought to set the stage for the kinds of processes that take place in ECEC settings. Process quality consists of the different interactions that children experience with staff and other children in their ECEC program. Thus, it is thought to impact children directly [13].

Staff education is a characteristic of structural quality that is often listed as an indicator of high-quality child care [14]. It is thought to be an important driver of the types of interactions and activities (e.g., level of cognitive stimulation) that children experience directly in ECEC programs, thereby influencing their outcomes. In keeping with the idea that structural quality drives process quality, there is evidence that higher levels of staff education are associated with higher quality interactions between staff and children [15]. However, in terms of findings about the impact of staff education on child outcomes results have been less consistent. While, a positive relationship between staff education and child outcomes has been reported in some studies [16,17], other studies did not find such associations [18–21].

Despite the lack of consistency in findings from studies that link staff education to child outcomes, education is still considered to be an influential quality indicator. For example, the American Academy of Pediatrics and the American Public Health Association recommend that early childhood educators have a minimum of a BA, a bachelor’s degree. They add that “at least 50% of all assistant teachers and teacher aides must have or be working on either a Child Development Associate (CDA) credential or equivalent, or an associate’s or higher degree in early childhood education/child development or equivalent.” (p. 12) [22]. This is consistent with recommendations by the National Association for the Education of Young Children [23] that early childhood educators should have a minimum of 4 to 5 years of post-secondary education. Finally, the UNICEF ‘Report Card 8’[24] sets 50% of staff having three years of higher education as the minimum quality benchmark for staff education in ECEC settings. Thus, major organizations in this sector highlight the importance of staff education and generally stipulate that staff working in ECEC settings should have undergraduate levels of education.

The perceived importance of staff education is further evidenced by the inclusion of staff education as part of Quality Ratings and Improvement Systems (QRISs), which are accountability systems used in the U.S. to monitor and improve ECEC program quality [25]. Programs receive a single amalgamated score based on number of characteristics (e.g., staff/child ratios, environmental rating scales etc.). In general staff education is one of the characteristics that is aggregated to create the program score.

Inconsistency in past findings makes it difficult to extract patterns of results from the literature on staff education. One reason for this is that education can be defined in different ways. Approaches to the measurement of staff education include: 1) years of education; 2) scales based on completed degrees; 3) defining thresholds or “levels” of education (e.g., BA/No-BA) and 4) rating participants in terms of whether or not they adhere to local quality standards for staff education. While these are sensible approaches, the variety in how education is operationalized makes it challenging to integrate findings across studies. In addition, ECEC classrooms are staffed by multiple adults. Different researchers can adopt different strategies with regards to whose education to include. For example, one strategy is to collect education information from different members of the staff in a classroom, another is to focus on the “lead” staff member (which can be an arbitrary distinction when ECEC programs adopt a team-teaching approach), and yet another is to simply collect education information for the staff member who is available when data collection takes place. Thus, while there is a growing body of research that examines the associations between staff education and child outcomes, it is difficult for individual stakeholders to extract conclusions or policy directives from this research. The complexities of this literature point to the need for a systematic review and meta-analysis of the association between staff education and child outcomes. To our knowledge such a review has not been published to date. Moreover, payment to staff is the major driver of costs in ECEC programs, and better-educated staff requires higher levels of remuneration. Thus, it is important to know whether there is a reliable relationship between ECEC staff education and child outcomes.

We set out to review whether higher levels of staff education are associated with better child outcomes. We explored whether there are more associations between staff education and child outcomes for at-risk children when compared to non at-risk children. Finally, we explored whether the pattern of associations differed when the education levels of multiple staff in a room were collected vs. those of the lead staff member only.

## Methods

### Search strategy

Electronic databases (PsycINFO, Medline, and ERIC) were searched for studies published until July 3, 2015. Two different search strategies were used to identify the eligible studies: 1) searching for education-related terms and child outcomes and, 2) a global approach that involved searching for a large number of quality indicators simultaneously. The second strategy allowed us to identify papers in which education was one of the control variables in the analyses. Thus, it ensured that we would capture studies that contribute to the current effort even if education was not the primary goal of the original study. Search terms are provided in Tables A-D in S1 File. The websites for the following large databases of ECEC quality and child outcomes were searched to locate research reports: Cost, Quality and Outcomes Study (CQO) [26]; Early Childhood Longitudinal Study (ECLS) [27]; Effective Provision of Pre-School Education (EPPE) Project [28]; Head Start Impact Study (HS) [29]; National Center for Early Development and Learning (NCEDL) Multi-State Study of Pre-Kindergarten [30]; State-Wide Early Education Program Study (SWEEP) [31]; Family and Child Experiences Survey (FACES) [32]; and the National Institute of Child Health and Human Development (NICHD) Study of Early Child Care and Youth Development [33]. Finally, reference sections of all retrieved studies were searched to locate additional studies. Finally, the search was limited to studies published in the English language only.

### Inclusion criteria

#### Types of studies

This review focused on cohort, cross-sectional and longitudinal studies reporting statistical associations between staff education in preschool classrooms to children’s academic competence (e.g., language and math) as well as cognitive, physical and social-emotional development outcomes. Studies that used a combination of staff education and other measures to create an overall quality composite that could not be disaggregated were excluded. (See Table 1 for a more detailed description of study selection criteria and rationale).

**Table 1:** Inclusion criteria for systematic review and rationale.

Criteria	Rationale
***Study Design***	
Cross-sectional and longitudinal designs were included. In some longitudinal studies child outcome data were collected at multiple time-points. When this happened, we used the data from the earliest time-point following the measurement of quality in our analyses.	To increase the homogeneity across the extracted data from eligible studies (i.e., increase the likelihood of meta-analysis), we focused on the earliest time-point in which child outcomes were measured following the measurement of quality in instances where multiple waves of outcome data were presented.
***Child Outcomes***	
Studies that provided information about the association between Staff Education on children’s academic competence (e.g., language and math) as well as cognitive, physical and social-emotional development outcomes were included. Data could have been gathered from teachers, parents, and/or children themselves. Measures that focus on dyads (e.g., attachment) were excluded.	Academic competence (e.g., language and math) as well as cognitive, physical and social-emotional development outcomes were selected because they are key predictors of children’s developmental trajectories. Measures that focus on staff-child or peer dyads were not included given that these outcomes often reflect an aspect of child care quality.
***Outcome Reporting***	
Studies must have presented statistical data quantifying the association between Staff Education and a child outcome measure.	Studies only reporting qualitative results were not considered for this review as the domains of assessment could vary markedly between studies.
***Language***	
To be extracted, studies had to be in English.	We did not have resources to systematically translate material written in other languages.
***Age Served***	
Studies that included preschool-aged children as the majority of participants were included. For the purposes of the meta-analysis, preschool-age was defined as ranging from 30 to 72 months.	Preschool-aged classrooms are different from infant/toddler classrooms due to the developmental stage and needs of the children in these two age groups. As a result, regulations and standards of care (e.g., ratios, physical environment, etc.), as well as daily activities (e.g., curriculum) differ between infant/toddler and preschool-aged classrooms.
***Child Care Type***	
Only studies that examined the impact of the quality of centre-based programs on children’s outcomes were included. Centre-based programs included daycare and preschool programs, nursery schools, pre-kindergarten programs, and Head Start programs. Studies that only examined home-based child care, or those in which home-based and centre-based could not be separated were excluded.	Center-based child care settings differ from home daycare in many ways such as ratios, group size, physical environment, curriculum, age range of children, and caregiver qualifications. As a result, quality is often measured differently for these two settings (e.g., ECERS^a^ versus FCCERS^b^).

Abbreviations: ECERS^a^ = Early Childhood Environment Rating Scale; FCCERS^b^ = Family Child Care Environment Rating Scale.

#### Types of participants and settings

We focused only on studies of preschool age children (30 to 72 months) as this age group serves the largest number of children and much of the research has been conducted on this age group [34,35]. Only the studies conducted in center-based ECEC settings were included (preschool, pre-kindergarten and Head Start programs, nursery schools, and child care centers). Studies examining only home-based child care or a mixture of home-based and center-based child care that could not be disentangled were not included.

### Outcomes

We cast a wide net regarding the child outcomes we included reflecting an understanding of the classroom context as having an impact on children that goes “beyond achievement tests” [36]. Thus, we compiled studies that used a broad range of outcomes and included any measure of academic competence (e.g., language and math) as well as cognitive, physical and social-emotional development outcomes. See Table 2 for a general description of each of the dimensions within the domains that were eligible for inclusion [37]. Also see S3 File for the list of the child outcome measures within each domain. The data in the reviewed studies were collected from staff, parents, and/or direct assessment of children. Measures that focus on dyads (e.g., attachment) were excluded as child and adult effects are difficult to disentangle in such measures.

**Table 2:** Outcome domains eligible for inclusion.

Domain	Descriptions of Dimensions
*Approach to Learning*	Children’s ability to adapt to and participate in the preschool environment including capacities such as initiative and curiosity, engagement and persistence, and reason and problem solving.
*Cognitive*	Aspects such as children’s readiness for learning, intellectual ability, and general knowledge.
*Combination*	Instruments that combine items across various domains such as developmental screeners.
*Language*	*Language Development* (speaking and communicating, listening and understanding) and *Literacy* (phonological awareness, book knowledge and appreciation, print awareness and concepts, early writing and alphabet knowledge).
*Mathematics*	Mastery of numbers and operations, geometry and spatial sense, and patterns and measurement.
*Physical Health & Development*	Gross motor skills, fine motor skills, and health status and practices.
*Social Emotional Behaviors*	Positive and negative behaviors, self-concept, self-control, cooperation, social relationships, knowledge of families & communities.

### Selection strategy

The selection of eligible studies was performed in two steps, each conducted by a pair of independent raters: 1) the titles and abstracts of the documents were screened for relevance; 2) full review of the relevant documents was conducted to determine if they met inclusion criteria for this study. In both steps pairs of trained raters included graduate students and authors of this paper (EM, OF and MP) in the Department of Applied Psychology and Human Development at the university of Toronto. Discrepancies between the raters were resolved through discussion or in consultation with the research methodologist (one of the authors–OF) who made the final decision. A systematic review protocol and data extraction form were developed by the research team and are available upon request from the first author.

### Data analysis

All studies that met our inclusion criteria were included in the systematic review. Subsets of studies that could be meta-analyzed together were also identified. In order to be meta-analyzed, studies had to report child outcomes measured with the same instrument and have staff education operationalized in the same way. While a meta-analysis can be conducted with as few as two studies [38] there is little empirical guidance in terms of the minimum number of studies required to conduct a meta-analysis. One source of information is a comprehensive review of all meta-analyses included in the Cochrane’s database, which found that the median number of studies included in meta-analyses was three [39]. Based on this finding we adopted three as the minimum for the number of studies required to conduct a meta-analysis in this review.

To increase homogeneity among studies that were meta-analyzed, only studies that ensured children’s exposure to the program were included. To do so, we only meta-analyzed studies that used child pre-scores as a covariate, used gain scores in analyses, or in which the authors stated explicitly that children had been in the program for a period of time prior to their assessment.

To avoid dependency issues when multiple studies were based on secondary analyses of subsamples drawn from the same dataset, only the study with the largest sample size was selected for inclusion in meta-analyses. [40]

For ethical and logistical reasons, all of the studies included in this review have a correlational/observational design, and, therefore, there is little variability in their scores on standard measures of study quality used for meta-analysis [41,42]. We did not proceed with these measures assessing study quality, as they did not allow us to differentiate between studies. In addition, due to sample size constraints we were not able to directly test for study quality as a moderator in our analyses. While we were not able to rate and test the impact of quality of study directly, we do note when papers were peer reviewed and provide readers with very detailed information about studies in the systematic review. Thus, we address the issue of study quality in this more qualitative way. It is worth noting that our selection criteria for the meta-analyses resulted in relatively stronger papers being included. This is because we required outcomes to have been used in three or more papers and this resulted in only the relatively psychometrically stronger measures being included. Only statistics that accounted for covariates (e.g., child and family characteristics) were combined within a single meta-analysis, which also pulled for the inclusion of the better quality research. Finally, when multiple studies drew from the same sample, we included the study with the largest sample size, which also pulled for the inclusion of studies with stronger methodologies.

For each meta-analysis, the *I*^*2*^ index was used to test statistical heterogeneity [43]. Large *I*^*2*^ values (>70%) indicate high heterogeneity in findings from different studies and reduce the reliability of the pooled results. Meta-analyses were performed using the random-effects models with the Comprehensive Meta-Analysis software (Version 3, see S2 File for conversion formulas) [44].

## Results

### Description of studies

**Fig 1:**
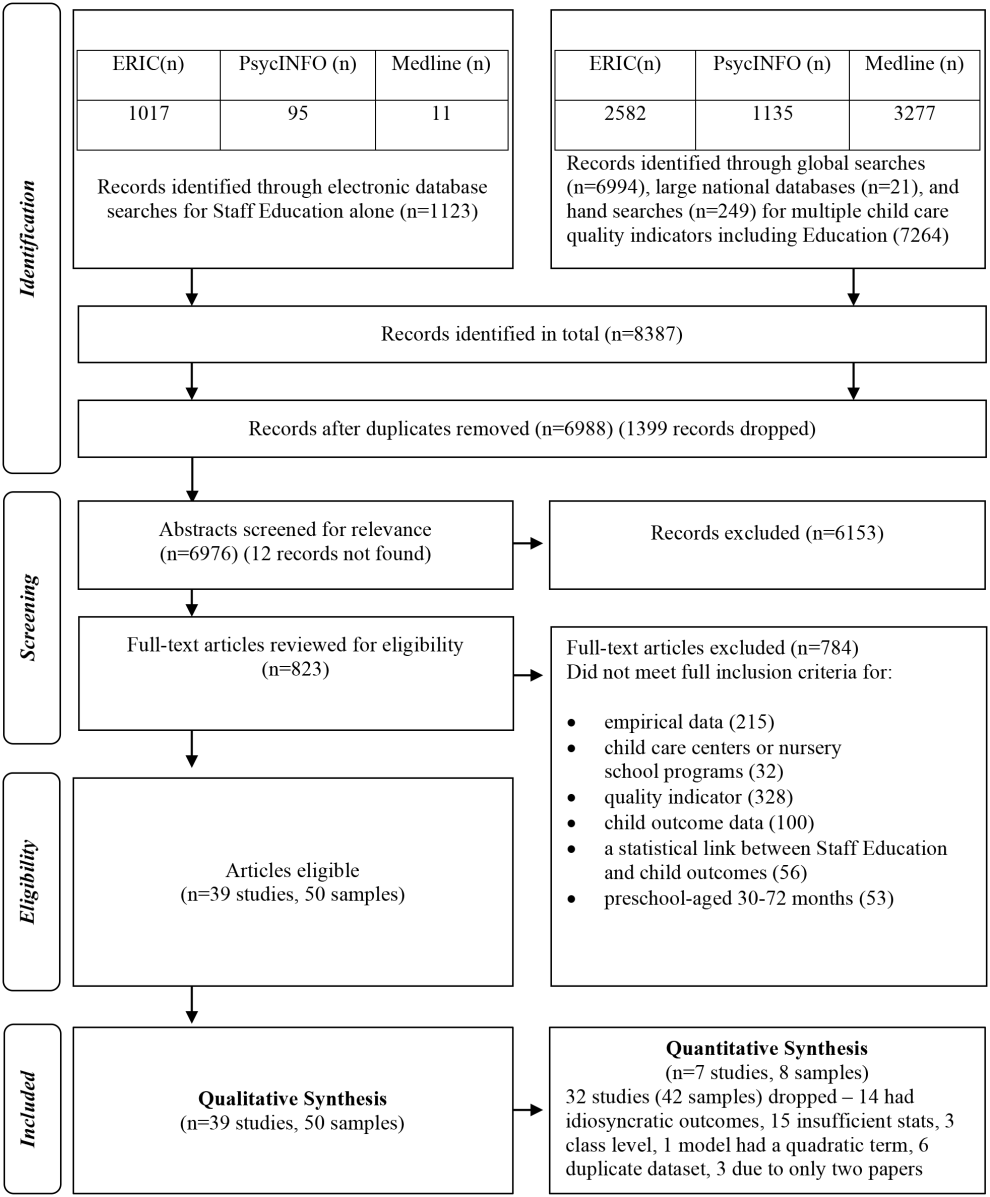
Flow diagram for study selection. Adapted from Moher et al. [45]

Results from the search and study selection are provided in Fig 1. Of the 823 potential studies related to the staff education, 784 were excluded because they did not meet all of the inclusion criteria. Thus, 39 studies were eligible for the current review. Of the 39 studies selected for this review, there are 26 peer reviewed journal articles, 10 reports and 3 books.

The characteristics of the 39 eligible studies are presented in Table 3 (the acronyms used in Table 3 can be found in S5 File). Thirty-four of the studies report the results for a single sample, 3 studies [19,46,47] provide the results for two samples of data, one study [48] has data for three samples of children, and one study [49] reports results from seven datasets of preschool programs. Table 3 contains a separate record for each of the 50 independent samples identified through our searches.

**Table 3:** Description of studies meeting inclusion criteria^a^.

Study^b^	Characteristics	Quality Measures M(SD)^c^	Outcome Measures M(SD)^d^	Covariates^e^
Aikens 2010[50]^m^^,^ ^B^ (2^nd^ doc. Hulsey 2010[51])	**Publication**: Report **Design**: Longitudinal **Dataset:** FACES 2006 **Country**: United States **Sample size:** classroom 410, children, range by analyses 2140–2931 **% Female:** 48.6 **Mean age:** 36–48 mo. **Ethnicity:** C23%, B33%, H35%, A2% **Mean maternal education**: NR **At High Risk >50% Children**: Yes	AA or Higher 39.45% Has a BA 38.34%	ECLS-Math 9.7 (3.19) PPVT-4 107.9 (16.27) Problem Behaviors 6.42 (0.26) SSRS-SS 17.3 (0.21) WJ-III-AP 390.3 (31.34) WJ-III-LWI 323.5 (25.76)	**Statistics Extracted:** Beta, Effect Size **Covariates:** child/family level—pretest score, age, gender, ethnicity, language, poverty, maternal education, maternal depressive symptoms, classroom level—full time classroom, peer social abilities, variation of peer abilities, peer abilities (PPVT), variation of peer abilities, DAP attitudes, staff education, program level–SES, % ELL, program curriculum package, teacher turnover, teacher salary
Aikens 2012[52]^M^ (2^nd^ doc. Moiduddin 2012[53])	**Publication**: Report **Design**: Longitudinal **Dataset:** FACES 2009 **Country**: United States **Sample size:** classroom 391, children, range by analyses 903–1936 **% Female:** 49.8 **Mean age:** 36–48 mo. **Ethnicity:** C45%, B66%, H73%, A3%, M11% **Mean maternal education**: NR **At High Risk >50% Children**: Yes	AA or Higher 34.7% Has a BA 40.7%	BPI-PB 3.9 (.20)—4.7 (.20) ECLS-B 49.1 (20.1)—66 (23.7) ECLS-K-PB 1.8 (0.0)—2.0 (0.1) EOWPVT 83.9 (14.3)—85.2 (14.8) PPVT 90.8 (14.6)—91 (15.) SS/CBS 16.5 (0.2)—17.9 (0.2) SS/CBS/PALS 12.2 (0.1)—12.6 (0.1) WJ-III-AP 91.2 (15.2)—93.6 (14.7) WJ-III-LWI 99.3 (14.4)—104.4 (19.1) WJ-III-S 97.4 (14.6) -97.5 (14)	**Statistics Extracted:** Effect Size **Covariates:** child/family level—pretest scores, child age at assessment, gender, ethnicity, language, household poverty ratio, maternal education, maternal depressive symptoms, time interval between the fall and spring assessments, program level—SES, percent DLLs, percent using curriculum and assessment from the same package, teacher turnover, program mean salary
Barnett 2007 [[46]Whole Sample Sample A: Spanish	**Publication:** Journal (ECRQ) **Design:** Longitudinal **Country:** United States **Sample size A:** classroom 36, children, range by analysis 128–131 **Sample size B:** classroom 36, children 74 **% Female:** 65.5 **Mean age:** NR **Ethnicity:** C7.5%, B13%, H76.3%, M2.3% **Mean maternal education:** NR **At High Risk >50% Children:** Yes	Ordinal (Has an MA/Not MA)	Alphabet Recognition-English (NR) Alphabet Recognition-Spanish (NR) WM-R-AP (NR) WM-R-PV (NR) Phoneme Deletion-English (NR) Phoneme Deletion-Spanish (NR) PPVT-III (NR) Rhyme Recognition-English (NR) Rhyme Recognition-Spanish (NR) TVIP (NR) WJ-R-AP (NR) WJ-R-PV (NR)	**Statistics Extracted:** B, SE **Covariates:** pretest score, age, gender, language, staff education (Has a MA), treatment group
Burchinal, Nelson 2000[54]^D^	**Publication:** Journal (ECRQ) **Design:** Longitudinal **Data Set:** CQO **Country:** United States **Sample size:** classroom NR, children 757 **% Female:** 48.9 **Mean age:** 48.4 **Ethnicity:** C67.9%, B15.9%, H4.6%, O11.6% **Mean maternal education:** 14.22 **At High Risk >50% Children:** NR	Years of Education	PPVT-R 93.59 (18.48)	**Statistics Extracted:** B, SE **Covariates:** state, ethnicity, gender, teacher responsiveness, child centered, CIS, group size, ratio, staff education
Burchinal, Roberts 2000[55]^T^	**Publication:** Journal (CD) **Design:** Longitudinal **Data Set:** OMS **Country:** United States **Sample size:** classroom 22, children 51 **% Female:** NR **Mean age:** 36 **Ethnicity:** B100% **Mean maternal education:** 12.5 years **At High Risk >50% Children:** Yes	College or Higher 14.9 (2.0)	Bayley-R-MDI 95.74 (10.15) SICD-RCA 33.4 (4.63) SICD-ECA 35.56 (4.53)	**Statistics Extracted:** Pearson’s Correlation **Covariates:** NA
Cameron 2011[18]	**Publication**: Journal (ECD) **Design**: Longitudinal **Country**: United States **Sample size:** classroom 41, children 140 (correlational analyses at classroom level) **% Female**: 54% **Mean age:** 51 mos. **Ethnicity:** C76%, B6%, H3%, O 15% **Mean maternal education**: 15.88 years **At High Risk >50% Children**: No	BA or Higher 25%	Alphabet Knowledge 17.15 (7,55) Emergent Literature Composite 0.00 (2.61) Head-to-toes Self-Regulation 12.11 (7.08) WJ III—AK 449.11 (14.96) WJIII—LWI 353.32 (25.50) WJ III—SA 457.83 (18.24) WJ III—PV 473.86 (11.90) WJ III—AP 419.62 (17.79)	**Statistics Extracted:** B, Pearson’s Correlation **Covariates:** child/family level—age, gender, Head Start, hours in preschool, maternal education, classroom level—staff education, group size, time spent orienting, interaction terms
Chang 2007[48] Whole Sample: NCEDL (Multi & SWEEP)^m^^,^^A^ Sample A: NCEDL (Spanish-Spanish Testing)^A^ Sample B: SWEEP (Spanish children)^A^	**Publication:** Journal (EED) **Design:** Longitudinal **Data Set:** NCEDL (SWEEP & Multi) **Country:** United States **Sample size A:** classroom 161, children 330 **Sample size B:** classroom 161, children 134 **Sample size C:** classroom 161, children 213 **% Female:** 52.17 **Mean age:** 55.32 **Ethnicity:** H100% **Mean maternal education:** NR **At High Risk >50% Children:** Yes	Ordinal (5 categories, (1) Some college only, (2) AA or 2 year, (3) BA, (4) At least 1 year beyond BA, (5) MA and above	PPVT-III 42.12 (11.76) Pre-LAS 17.49 (12.45) TVIP 22.4 (11.74)	**Statistics Extracted**: B, SE, Beta **Covariates:** child/family level—ethnicity, income, maternal education, classroom level–staff education, proportion Latino peers, teacher-child closeness, teacher-child language interaction
Choi 2014[56]	**Publication**: Journal (ECDC) **Design**: Longitudinal **Country**: United States **Sample size:** classroom 31, children 129 **% Female:** 43.45 **Mean age:** NR **Ethnicity:** C58.1%, H10,1%, B 6.2%, A14.0%, M10.1%, O 1.6% **Mean maternal education**: No HS/GED 5.4%, HS/GED 10.1%, some college/post-HS 15.5%, AA 10.1%, BA or Higher 58.9% **At High Risk >50% Children**: No	Has a BA 51.6%	TEMA-3–5.84(0.69)	**Statistics Extracted:** Beta **Covariates:** child/family level—pretest score, fall test age, gender, ethnicity, parent education (BA), duration between test days, previous childcare experience, hours in care T-C closeness x teaching experience, classroom level activities (a) counting, (b) basic operations, (c) shape and pattern, (d) measurement, (e) geometry, child care subsidy
Clarke-Stewart 1994[57]	**Publication:** Book **Design:** Longitudinal **Country:** United States (Chicago) **Sample size:** classroom NR, children 62 **% Female:** NR **Mean age:** 37 months **Ethnicity:** C85%, B12%, A2%, H1% **Mean maternal education:** NR **At High Risk >50% Children:** No	Ordinal 5.1 (1.7) (6 point scale ranging from 1 = Junior High to 6 = Post graduate degree)	Compliance w/ Parents (NR) Compliance w/ Requests (NR) General Compliance at Home (NR) Intellectual Ability (NR) Social Cognitive Ability (NR) Social Comp. w/ Stranger (NR) Social Comp. w/ Visitor (NR)	Statistics Extracted: Partial Correlation Covariates: age
Colwell 2013[58]^,^^N^	**Publication**: Journal (ECRQ) **Design**: Longitudinal **Dataset:** Early Childhood Longitudinal Study-Birth Cohort (ECLS-B) **Country**: United States **Sample size:** classroom NR, children 1000 **% Female:** 49 **Mean age:** NR **Ethnicity**: Hispanic 24%, Non-Hispanic Black 16% Non-Hispanic White 53%, Non-Hispanic Other 7% **Mean maternal education**: NR **At High Risk >50% Children**: NR	Years of Education M(SD) 17.31 (2.00)	ECLS Math -0.33 (.78) ECLS Literacy -0.37 (0.73) **Parent Report** Social Competence 4.00 (0.55) Emotional/Behavioral Reg. 2.42 (0.47) Attention and Concentration 2.91 (0.40) **Caregiver Report** Social Competence 3.78 (0.58) Emotional/ Behavioral Reg. 1.88 (0.69) Attention and Concentration 2.70 (0.50)	**Statistics Extracted:** Beta **Covariates:** child level—pretest score, gender, ethnicity, low birth weight, breastfed, well-child doctor visits, received WIC (a) health 9 mo. & 2 yr., (b) absence of common illness 9 mo. & 2 yr., (c) temperament 9 mo. & 2 yr., absence of injury, family level–maternal (a) birthplace not US, (b) other child ages < 6, (c) other children 6–18 years, (d) employment status, (e) marital status, (f) age, family received (a) TANF, (b) food stamps, community level–region, urbanicity, centre level–provider’s (a) gender, (b) age, (c) ethnicity, (d) experience, (e) ECE certificate, hours per week in care, months in care, center (a) type, (b) accreditation status, (c) license status for what size, (d) center accepts subsidies (11)
Dotterer 2013[59]^A^	**Publication**: Journal (ECDC) **Design**: Longitudinal **Dataset:** NCEDL (Multi-State & SWEEP) **Country**: United States **Sample size:** classroom 716, children 3584 **% Female:** 51.17 **Mean age:** 48 mo. **Ethnicity:** C41%, B18%, H27%, O14% **Mean maternal education**: 12.62 years **At High Risk >50% Children**: $36,041	Years of Education M(SD) 15.94 (1.73)	Acad. Rat. Scale 92.22 (0.93) Naming Letters 6.2 (3.65) Naming Numbers 11.71 (9.33) OWLS-Exp. Lang 90.61 (12.24) PPVT-III 92.22 (13.32) WJ-III-Rhyming 2.76 (3.43) WJ-III-AP 96.11 (12.26)	**Statistics Extracted:** B, SE **Covariates:** child/family level—gender, ethnicity, maternal education, classroom level—hours per day, % Caucasian, poverty, program, poverty x program, staff education, staff-child ratio, ECERS—Language & Interaction, ECERS—Provision for Learning
Downer 2012[19] Sample A: Dual Language Learners^A^ Sample B: Latino^A^	**Publication**: Journal (ECRQ) **Design**: Longitudinal **Dataset:** NCEDL (Multi-State & SWEEP) **Country**: United States **Sample size:** classroom 721, children **Sample A** 956, **Sample B** 328 **% Female**: 51 **Mean age:** NR **Ethnicity:** C40%, B18%, H26%, O16% **Mean maternal education**: 12.6 years **At High Risk >50% Children**: Yes	Has a BA 66%	**Whole Sample** ARS-Language 3.0 (0.97) TCRS-SS 0.77 (3.64) TCRS-PB 1.57 (0.75) WJ-III-AP 412.19 (18.88) WJ-III-LWI 12.9 (9.61)	**Statistics Extracted:** B, SE, T-Test **Covariates:** child/family level—pretest score, gender, ethnicity, maternal education, poverty, language, test interval, test in Spanish, DLL status, classroom level—staff education (BA), staff-child ratio, poverty, full-day, state, teacher/teacher’s assistant speaks Spanish, percent DLL, staff-child ratio, staff education, interaction terms
Dunn 1993[60]]^S^	**Publication:** Journal (ECRQ) **Design:** Longitudinal **Data Set:** NCEDL **Country:** United States **Sample size:** classroom 30, children 60 **% Female:** 51 **Mean age:** 51.85 **Ethnicity:** B60% **Mean maternal education:** 13.4 years **At High Risk >50% Children:** No	Years of Education M(SD): 14.57 (2.17) Ordinal (7 levels) (0) none, (1) HS course, (2) Jr. College/ Technical School courses or CDS training, (3) AA, (4) BA, (5) MA, (6) PhD)	CBI-Intellectual 53.88 (20.24) CBI-Preschool 33.87 (15.67) CBQ 13.78 (8.96) PSI-R 44.8 (9.2)	**Statistics Extracted:** Pearson’s Correlation, Partial Correlation **Covariates**: SES, maternal education, income, experience-centre, degree
Early 2006[34]]^A^	**Publication**: Journal (ECRQ) **Design**: Longitudinal **Dataset:** NCEDL (Multi-State) **Country**: United States **Sample size:** classroom 237, children, range by analyses 714–845 **% Female:** 51 **Mean age:** 54.7 mo. **Ethnicity:** C41%, B24%, H25%, A2%, M8% **Mean maternal education**: NR **At High Risk >50% Children**: Yes	Years of Education M(SD): 15.67 (2.07) BA or Higher Ordinal (4 levels (1) no degree, (2) AA, (3) BA, (4) more than a BA)	Identifying Colors 9.29 (1.73) Identifying Letters 12.26 (9.5) dentifying Numbers 6.26 (3.67) OWLS-Oral Exp. 94.79 (12.29) PPVT-III 95.69 (13.58) WJ-III-AP 98.56 (11.86) WJ-III-SA 2.95 (3.54)	**Statistics Extracted:** B, SD, Pearson's Correlation, Partial Correlation, Adjusted Means, Cohen’s D **Covariates:** state, fall scores, program in school, hours per week, maternal education, staff-child ratio, staff education, ECERS
Early 2007[49] Sample A: Head Start^H^	**Publication**: Journal (CD) **Design**: Longitudinal **Dataset:** EHS **Country:** United States **Sample size:** classroom NR, children 887 **% Female**: 50% **Mean age:** 37.1 mos. **Ethnicity:** C37%, B36%, H25%, O3% **Mean maternal education**: NR **>50% Children**: NR	Has a BA Ordinal (4 categories, (1) HS or GED, (2) AA, (3) BA, (4) Graduate degree)	PPVT-III 92.31 (14.44) WJ-R: L-W ID 90.38 (15.03) WJ-R: AP 88.31 (17.85)	**Statistics Extracted:** F-Ratio, Cohen's d **Covariates:** gender, ethnicity, poverty, pretest scores, maternal education, Head Start(Y/N), classroom size, school day hours, teacher's ethnicity
Early 2007[49] Sample B: FACES 2003^L^	**Publication**: Journal (CD) **Design**: Longitudinal **Dataset:** FACES 2003 **Country: United States** **Sample size:** classroom 310, children 1041 **% Female**: 51% **Mean age:** 47.98 mos. **Ethnicity:** C23%, B35%, H32%, O10% **Mean maternal education**: NR **At High Risk >50% Children**: Yes	Has a BA Ordinal (4 categories, (1) HS or GED, (2) AA, (3) BA, (4) Graduate degree)	PPVT-III 86.19 (11.68) WJ-R: LWI 99.86 (15.62) WJ-R: AP 92.58 (14.16)	**Statistics Extracted:** F-Ratio, Cohen's d **Covariates:** gender, ethnicity, poverty, pretest scores, maternal education, ratios, classroom size, school day hours, teacher's ethnicity
Early 2007[49] Sample C: GECS 2002^F^	**Publication**: Journal (CD) **Design**: Longitudinal **Dataset:** GECS 2002 **Country: United States** **Sample size:** classroom 138, children 630 **% Female**: 47% **Mean age:** 55.5 mos. **Ethnicity:** C49%, B40%, H2%, O9% **Mean maternal education**: NR **At High Risk >50% Children**: Yes	Has a BA Ordinal (4 categories, (1) HS or GED, (2) AA, (3) BA, (4) Graduate degree)	PPVT-III 96.56 (14.5) WJ-III: LWI 103.77 (13.37) WJ-III: AP 98.3 (13.31)	**Statistics Extracted:** F-Ratio, Cohen's d **Covariates:** gender, ethnicity, poverty, pretest scores, maternal education, program type (GA Pre-K, Head Start, private), ratios, classroom size, school day hours, Caucasian, teacher's ethnicity, classroom poverty
Early 2007[49] Sample D: MAF 2002–2004^YA^^,^^YB^	**Publication**: Journal (CD) **Design**: Longitudinal **Dataset:** MAF 2002–03 and 2003–04 **Country: United States** **Sample size:** classroom 98, children 785 **% Female**: 51% **Mean age:** 54 mos. **Ethnicity:** C35%, B43%, H15%, O8% **Mean maternal education**: NR **At High Risk >50% Children**: Yes	Has a BA Ordinal (4 categories, (1) HS or GED, (2) AA, (3) BA, (4) Graduate degree)	PPVT-III 89.57 (16.2) WJ-III: AP 93.98 (13.24)	**Statistics Extracted:** F-Ratio, Cohen's d **Covariates:** gender, ethnicity, poverty, pretest scores, school year, ratio, classroom size, Caucasian, MAF
Early 2007[49] Sample E: NCEDL^m^^,^^A^	**Publication**: Journal (CD) **Design**: Longitudinal **Dataset:** NCEDL **Country: United States** **Sample size:** classroom 721, children 2966 **% Female**: 51% **Mean age:** 55.4 mos. **Ethnicity:** C41%, B18%, H26%, O14% **Mean maternal education**: NR **At High Risk >50% Children**: Yes	Has a BA Ordinal (4 categories, (1) HS or GED, (2) AA, (3) BA, (4) Graduate degree)	PPVT-III 96.29 (14.31) WJ-III: AP 99.11 (12.85) WJ-III: LWI 102.92 (14.08)	**Statistics Extracted:** F-Ratio, Cohen's d **Covariates:** gender, ethnicity, poverty, pretest scores, maternal education, public school, full-day, ratio, classroom size, school day hours, Caucasian, teachers' ethnicity, classroom poverty
Early 2007[49] Sample F: NICHD^m^^,^ ^Q^	**Publication**: Journal (CD) **Design**: Longitudinal **Dataset:** NICHD **Country: United States** **Sample size:** classroom 639, children 639 **% Female**: 50% **Mean age:** 36 mos. **Ethnicity:** C80%, B10% H5%, O5% **Mean maternal education**: NR **At High Risk >50% Children**: No	Has a BA Ordinal (4 categories, (1) HS or GED, (2) AA, (3) BA, (4) Graduate degree)	PLS-3: Auditory Comp. & Expressive Lang. 101.2 (19.79) WJ-R: L-W ID 100.59(13.39) WJ-R: AP 105.06 (15.22)	**Statistics Extracted:** F-Ratio, Cohen's d **Covariates:** gender, ethnicity, poverty, pretest scores, maternal education, ratios, classroom size, school day hours, teacher's ethnicity
Early 2007[49] Sample G: PCER^U^	**Publication**: Journal (CD) **Design**: Longitudinal **Dataset:** PCER **Country: United States** **Sample size:** classroom 76, children 667 **% Female**: 48% **Mean age:** 56.17 mos. **Ethnicity:** C30%, B44%, H18%, O9% **Mean maternal education**: NR **At High Risk >50% Children**: Yes	Has a BA Ordinal (4 categories, (1) HS or GED, (2) AA, (3) BA, (4) Graduate degree)	PPVT-III 93.78 (14.42) WJ-III: LWI 101.73 (14.3) WJ-III: AP 97.32 (13.8)	**Statistics Extracted:** F-Ratio, Cohen's d **Covariates:** gender, ethnicity, poverty, pretest scores, maternal education, ratios, classroom size, teacher's ethnicity
Epstein 1993[61]	**Publication**: Book **Design**: Longitudinal **Dataset:** High/Scope **Country**: NR **Sample size:** classroom 26, children 200 (analyses at program level n = 26) **% Female**: 53.5% female **Ethnicity:** NR **Mean maternal education**: NR **At High Risk >50% Children**: NR	Years of Education	COR-Total (NR) COR-Logic/Math (NR) COR-Representation (NR) COR-Language (NR) COR-Initiative (NR) COR-Social (NR) COR-Music (NR) DIAL-R-Total (NR) DIAL-R-Math (NR) DIAL-R-Concepts (NR) DIAL-R-Language (NR)	**Statistics Extracted:** Pearson’s Correlation **Covariates:** none
Guo 2014[62]^U^	**Publication**: Journal (EED) **Design:** Longitudinal **Dataset:** PCER **Country**: United States **Sample size:** classroom 16, children 130 **% Female:** 45 **Mean age:** 53.76 mo. **Ethnicity:** C72%, B21%, H4% **2014 Mean maternal education**: NR **At High Risk >50% Children**: No	Has a BA Ordinal (4 levels, (1) some college 12.5%, (2) AA 12.5%, (3) BA 50%, (4) MA) 25%	PPVT-III 68.94 (15.68)	**Statistics Extracted:** B **Covariates:** child/family level—pretest score, age, gender, family income, classroom age SD, interaction terms
Hamre 2014[63]	**Publication**: Journal (CD) **Design:** Longitudinal **Dataset:** Sample Hamre et al., 2012 **Country**: United States **Sample size:** classroom 314, children 1407 **% Female:** 51 **Mean age:** 4.17 years **Ethnicity:** B47%, H34%, C11.4%, A2.4%, O5.2% **2014 Mean maternal education**: NR (generally low) **At High Risk >50% Children**: Yes	Years of Education	Backward Digit Spin- 1.35 (.69) Pencil Tap .64 (.33) PPVT-III 50.59 (19.51) STRS-Closeness 4.49 (.58) STRS-Conflict 1.80 (.92) TOPEL-PA 14.88 (5.57) TOPEL-PK 21.42 (11.32) WJ-PV 13.61 (3.66)	**Statistics Extracted:** B, SE **Covariates:** child/family level—pretest score, age, gender, ethnicity, days between assessments, maternal education, intervention group, classroom level—staff education, teacher experience, income to needs, Head Start, Public School, curriculum
Henry, Gordon 2003[64]^I^	**Publication**: Report **Design:** Longitudinal **Dataset::** Georgia Pre-K **Country**: United States **Sample size:** classroom 203, children 2389 **% Female:** 49 **Mean age:** NR **Ethnicity:** C50%, B39%, A3%, H5%, M2%, O1% **Mean maternal education**: NR **At High Risk >50% Children**: Yes	College or Higher BA or Higher	Retention rates (NR) Stanford 9: Math 46.3 (NR) Stanford 9: Language Arts 47.8 (NR) Stanford 9: Science 46.7 (NR) Stanford 9: Social Studies 47 (NR)	**Statistics Extracted:** B **Covariates:** gender, ethnicity, Risk variable (parental education, household income, means tested federal program use), quality X Risk
Henry 2005[65]^F^	**Publication:** Report **Design:** Longitudinal **Data Set:** GECS **Country:** United States **Sample size:** classroom NR, children 670 **% Female:** 48.3 **Mean age:** 54 **Ethnicity:** C57.7%, Black = 33%, H3.6%, O4.9% **Mean maternal education:** NR **At High Risk >50% Children:** No	Has a BA	PPVT-III 106 (12.3) Story & Print 7.1 (2.6) WJ-III-AP 100.1 (13.3)	**Statistics Extracted:** B **Covariates:** child/family level–pretest score, gender, ethnicity, income, subsidy, lived continuously with both parents, maternal education, classroom level–ability, % male, ethnicity, time spent on discipline, program type, group size, teacher’s experience, staff education (Has a BA)
Hindman 2010[20]^J^	**Publication:** Journal (ECRQ) **Design:** Longitudinal **Data Set:** FACES 1997 **Country:** United States **Sample size:** classroom NR, children 945 **% Female:** 44.5 **Mean age:** 51.94 **Ethnicity:** C32%, B25%, A2%, H33%, M7%, AI = 2% **Mean maternal education:** 3.08 years **At High Risk >50% Children:** Yes	Ordinal (10-point scale) 7.02 (1.38) From 8th grade or less to completed graduate degree. A value of 7 represents some additional college coursework beyond an associate’s degree but no bachelor’s degree	WJ/WM-D 449.83 (19.67) WJ/WM-AP 461.42 (16.66)	**Statistics Extracted:** B **Covariates:** child/family level language skills, social skills, ethnicity, gender, age, disability diagnosis, parent involvement, maternal education, mastery, poverty/ public assistance status, classroom level—teacher background, classroom size, affective quality, structural features of the center, teacher experience, staff education
Howes 2008[66]^A^	**Publication:** Journal (ECRQ) **Design:** Longitudinal **Data Set:** NCEDL & SWEEP **Country:** United States **Sample size:** classroom 70, children, range by analysis 1787–2044 **% Female:** 51 **Mean age:** NR **Ethnicity:** C42%, O58% **Mean maternal education:** 12.8 years **At High Risk >50% Children:** Yes	Has a BA	Identifying Letters (NR) Language/Literacy (NR) OWLS-Oral Exp. (NR) PPVT-R (NR) WJ-III-AP (NR) SSRS-SS (NR) SSRS-BP (NR)	**Statistics Extracted:** B, SE, Pearson’s Correlation **Covariates:** child/family level—state, gender, child age at fall assessment, ethnicity, maternal education, poverty, number of people in the household, classroom level—staff education (BA), ratios, in/out school, full/part-day, T-C relationship, CLASSROOM Emotional Climate, CLASSROOM Instructional Climate, ECERS-R Provisions for Learning for learning
Kaiser 2002[67]	**Publication**: Journal (BD) **Design**: Cross-Sectional **Country:** United States **Sample size:** classroom 14, children 332 **% Female:** 49% **Mean age:** 42 mos. **Ethnicity:** B88% **Mean maternal education**: 12 **At High Risk >50% Children**: Yes	Ordinal (2 level) (1) AA 65%, (2) BA 35%	(NR)	**Statistics Extracted:** B **Covariates:** gender, pretest scores, teacher experience
Kim 2011[16]^L^	**Publication**: Journal (CYSR) **Design**: Longitudinal **Dataset**: FACES 2003 **Country**: Unites States **Sample size:** classroom 409, children 2297 (weighted) **% Female**: 52.3% **Mean age**: 48 mos. **Ethnicity:** H95%, O 5% **Mean maternal education**: 3.4 (On scale equivalent to GED) **At High Risk >50% Children**: NR	BA or Higher <BA 62.2%, BA+ 37.8%	WJ R—AP 87.73 (16.64)	**Statistics Extracted:** Pearson’s Correlation **Covariates:** none
Lyon 1995[68]	**Publication:** Report **Design:** Cross-Sectional **Data Set:** Atlantic Day Care Study **Country:** Canada **Sample size:** 48 centers, classroom NR, children, range by analysis 489 **% Female:** 50 **Mean age:** 47.53 **Ethnicity:** NR **Mean maternal education:** NR **At High Risk >50% Children:** No	Ordinal (3 categories) (1) HS, (2) College, (3) University	ALI 57.1 (12.24) PPVT 97.68 (14.94) Entwistle Scale 73.06 (14.72) PSPCSAYC-Peer Acceptance 3 (0.64) PSPCSAYC-Maternal Acceptance 3.11 (0.6) PSPCSAYC-Physical Competence 3.23 (0.5) PSPCSAYC-Cognitive Competence 3.58 (0.47)	**Statistics Extracted:** F-Ratio **Covariates:** none
Mashburn 2004[17]^F^	**Publication**: Journal (EMIP) **Design**: Cross-Sectional **Dataset**: ECS **Country**: United States **Sample size:** classroom NR, children 406 **% Female**: 48% female **Mean age:** NR **Ethnicity:** C54%, B36%, O12% **Mean maternal education**: No **At High Risk >50% Children**: NR	Ordinal (3 categories) (1) less than BA, (2) BA, (3) Advanced Degree	SRF: Academic factor (NR) SRF: Comm. Skills factor (NR) SRF: Kindergarten readiness (NR)	Statistics **Extracted**: Beta **Covariates:** pretest scores, age, gender, ethnicity, mothers' education, family instability, welfare status, program type
Mashburn 2010[21]^W^	**Publication**: Journal (ADP) **Design**: Longitudinal **Country**: United States **Sample size:** classroom 134, children 1165 **% Female**: 49% **Mean age**: 50.4 mos. **Ethnicity:** NR **Mean maternal education**: 12.7 years **At High Risk >50% Children**: Yes	Ordinal (2 level) (1) BA, (2) Advanced Degree	PALS: Emergent Literacy 60.2 (12.6) Pre-CTOPP: Blending Sounds 8.29 (2.88) Pre-CTOPP: Elision 7.43 (3.41) Pre-CTOPP: Print Awareness 28.9 (7.6) Pre-CTOPPP: Receptive Vocab 34.1 (3.41)	**Statistics Extracted:** B, SE **Covariates:** child/family level—pretest score, gender, ESL, maternal education, poverty, year of intervention, classroom level—pretest score, percent poverty, percent ESL, maternal education, staff education, teacher experience in preK, intervention status
Mashburn, Pianta 2008[69]^A^	**Publication:** Journal (CD) **Design:** Longitudinal **Data Set:** NCEDL & SWEEP **Country:** United States **Sample size:** classroom 671, children, range by analysis 2307–2439 **% Female:** 51 **Mean age:** NR **Ethnicity:** C46%, B21%, H27%, O15% **Mean maternal education:** 12.9 years **At High Risk >50% Children:** No	BA or Higher	Letter Naming 13.9 (9.42) OWLS-Oral Exp. 93.6 (13) PPVT-III 96.3 (14.3) TCRS-SS 3.66 (0.7) TCRS-PB 1.49 (0.54) WJ-III-SA 3.65 (4.02) WJ-III-AP 99.1 (12.9)	**Statistics Extracted:** B, SE **Covariates:** pretest scores, gender, ethnicity, mother’s education, poverty, state
Montie 2006[70]	**Publication**: Journal (ECRQ) **Design**: Longitudinal **Dataset:** IEA **Country**: International Sample **Sample size:** classroom 426, children 1300 **% Female**: NR **Mean age:** 54–57.6 mos. **Ethnicity:** NR **Mean maternal education**: NR **At High Risk >50% Children**: No	Years of Education M(SD): 13.9 (2.79)	Cognitive 0 (1) Language 0 (1)	**Statistics Extracted:** B, SE **Covariates:** child/family level—pretest score, age, gender, number of siblings, parent education, country level—adult teaching, pre-academic, adult-child interaction, classroom level—age spread, mean parent education, whole group activity, materials
NICHD ECCRN 1999[71]^Q^	**Publication:** Journal (AJPH) **Design:** Longitudinal **Dataset:** NICHD **Country:** United States (National) **Sample size:** classroom NR, children range by analyses 49–250 **% Female:** NR **Mean age:** 36 months **Ethnicity:** NR **Mean maternal education:** NR **At High Risk >50% Children:** No	College or Higher 3.24 (0.91)	Bracken School Readiness (NR) Reynell Scales Expressive (NR) Reynell Comprehension (NR) Behavior Problems Composite (NR) Positive Social Behavior Composite (NR)	**Statistics Extracted:** Adjusted Means, SE, F-Ratio **Covariates:** ratio of income to needs, maternal sensitivity
Reid 2013[72]^m^^,^ ^A^	**Publication**: Journal (EED) **Design:** Longitudinal **Dataset:** NCEDL (Multi-State & SWEEP) **Country**: United States **Sample size:** classroom 704, children 2966 **% Female:** NR **Mean age:** NR **Ethnicity:** NR **2014 Mean maternal education**: 12.8 **At High Risk >50% Children**: Yes	Has a BA BA or Higher Low/Mod/High SES No BA 37%/26%/23% More than BA 28%/25%/20% Ha BA 35%/49%/47%	Hightower (NR) OWLS-Oral Exp. (NR) PPVT (NR) WJ- III AP (NR)	Statistics **Extracted:** Beta **Covariates:** child/family level—pretest score, gender, age, SES, ethnicity, single parent, ELL status, IEP status, classroom level—SES, deviation of income, percent Caucasian, teacher has BA, teacher has more than a BA, classroom size (less than 18), full-day, Head Start, interaction terms
Research Triangle Institute 1972[47] Study A	**Publication**: Report **Design**: Longitudinal **Dataset**: HS 1967–1968 **Country**: United States **Sample size:** classroom 177, children 1889 **% Female**: 49.3% female **Ethnicity:** C32%, B50%, O18% **Mean maternal education**: NR **At High Risk >50% Children**: Yes	Ordinal (4 categories) (1) HS 5%, (2) some college 24% (3) AA or Higher 10%, BA/BS 37%, BA/BS + course 19%, (4) MA or Higher 5%	Stanford Binet 4.69 (10.05) Behaviour Problem -0.15 (1.16) Motivational Problem -0.56 (2.26) Feeling of Inadequacy -0.31 (1.34)	NR
Research Triangle Institute 1972[47] Study B	**Publication**: Report **Design**: Longitudinal **Dataset**: HS 1968–1969 **Country**: United States **Sample size:** classroom 148, children 1443 **% Female**: 49.5% female **Ethnicity:** C18%, B68%, O14% **Mean maternal education**: NR **At High Risk >50% Children**: Yes	Ordinal (4 categories) (1) HS, (2) some college, (3) 2–4 year degree, (4) beyond 4 year	PSI 9.38 (7.73) Stanford Binet 4.82 (10.19) WPPSI 9.45 (12.77) GUMP 7.55 (7.98) Behaviour Problem -0.09 (1.31) Motivational Problem -0.21 (2.42) Feeling of Inadequacy -0.20 (1.36)	**Statistics Extracted:** NR **Covariates:** NR
Sabol 2013[73]^m^^,^^A^	**Publication**: Journal (EED) **Design**: Longitudinal **Data Set:** NCEDL (Multi-State and SWEEP) **Country**: United States **Sample size:** classroom 673, children 2419 **% Female:** 47 **Mean age:** 4.61 **Ethnicity:** C42%, B25%, H18%, O15% **Mean maternal education**: 12.96 years **At High Risk >50% Children**: No	Has a BA Ordinal (Has MA/Not MA) Ordinal (5 categories) (1) HS or less 8%, (2) CDA 7%, (3) AA 12%, (4) BA 47%, (5) MA+ 24%	Letter Knowledge 14.40 (9.34) OWLS-Oral Exp. 93.21 (13.45) PPVT-III 95.52 (14.70) TCRS-Problem Behaviors 1.49 (0.55) TCRS-Social Skills 3.56 (0.77) WJ AP 98.88 (13.37) WJ Rhyming 3.36 (3.82)	**Statistics Extracted:** Pearson’s Correlation, Beta **Covariates:** child/family level—pretest score, gender, ethnicity, maternal education, poverty, household size, attend pre-k prior year, classroom level—state, ethnicity, Head Start
Son 2013[74]^m^^,^^L^	**Publication**: Journal (CYCF) **Design**: Longitudinal **Dataset:** FACES 2003 **Country**: United States **Sample size:** classroom 310, children 2,159 **% Female:** 49.1 **Mean age:** NR **Ethnicity:** C 28.7%, B34.6%, H31.3%, O1.3% **Mean maternal education**: NR **At High Risk >50% Children**: NR	Ordinal (4 levels) (1) HS, 6.5%, (2) AA 57.4%, (3) BA 27.7%, (4) MA 7.7%	WJ-R-LWI WJ-R-AP Teacher-Reported Social skills PPVT-III PLBS PSSPAL	**Statistics Extracted**: Pearson’s Correlation, Beta **Covariates:** child/family level—pretest score, gender, ethnicity/minority status, maternal education, home language/assessment in Spanish, classroom level—social-emotional practices, provisions for learning, parental involvement practices, teacher (a) education, (b) experience, (c) teaching certificate, (d) specialized training, (e) coaching support
Travers 1980[75]	**Publication:** Report **Design:** Longitudinal **Dataset:** NDSC **Country:** United States (urban areas sample) **Sample size:** classroom 117, children 1383 (analyses at center level n = 54–57) **% Female:** NR **Mean age:** 36 and 48 months **Ethnicity:** C30%, B65%, O5% **Mean maternal education:** 59% High school or less **At High Risk >50% Children:** No	Years of Education M(SD): 13 years 10 months (15 months) Ordinal (NR)	PPVT (NR) PSI (NR)	**Statistics Extracted:** B, Pearson Correlation, F-Ratio **Covariates:** NR
West 2010[76]^B^ (2^nd^ doc. Malone 2010[77])	**Publication**: Report **Design**: Longitudinal **Dataset:** FACES 2006 **Country**: United States **Sample size:** classroom 410, children, range by analyses 426–684 **% Female:** 49 **Mean age:** 36–48 mo. **Ethnicity:** C25%, B27%, H39%, A2%, M5%, O3% **Mean maternal education**: NR **At High Risk >50% Children**: Yes	BA or Higher	ECLS-Math 19 (NR) PPVT-4 95 (NR) SSRS-BP 6.7 (NR) SSRS-SS 18 (NR) WJ-III-LWI 334.5 (NR) WJ-III-AP 401.5 (NR)	**Statistics Extracted:** Beta **Covariates:** child/family level—child's exposure to HS (1 vs. 2 years), gender, ethnicity, language, poverty ratio, joint book reading at least 3 times per week, number of books in home, maternal education, parent depressive symptoms, Low/mid/High ability at HS entry, classroom level—mean peer abilities at HS entry on WJ-AP, variation in peer abilities at HS entry on WJ-AP, full day/half day, ECERS—Teaching and Interactions, ECERS—Provisions for Learning for Learning, staff education (Has a BA)
Zellman 2008[78]^m^^,^ ^Z^	**Publication:** Report **Design:** Longitudinal **Country:** United States **Sample size:** classroom 156, children 1368 **% Female:** 50 **Mean age:** 47.338 **Ethnicity:** NR (42% minority) **Mean maternal education:** NR **At High Risk >50% Children:** No	Has a BA	CBI-Apathy 2.134 (0.733) CBI-Considerateness 3.489 (0.868) CBI-Creativity 3.737 (0.773) CBI-Dependence 2.447 (0.806) CBI-Distractibility 2.581 (0.87) CBI-Independence 3.79 (0.682) CBI-TO 3.431 (0.872) CBI-Verbal 3.507 (0.879) PPVT-III 92.756 (14.89) WJ-AP 97.419 (14.392) WJ-LWI 104.755 (16.728) WJ-PC 115.707 (13.322)	**Statistics Extracted:** B, SE **Covariates:** child/family level—age at assessment, gender, learning problems, hours per week with provider, duration with provider, family income, maternal education (has a BA), minority status, speaks other language besides English, parents' child-rearing style, classroom level—Head Start program, nonprofit organization, level of intervention intensity as determined by Qualistar
Zill 2003[79]^K^	**Publication:** Report **Design:** Longitudinal **Data Set:** FACES 2000 **Country:** United States **Sample size:** classroom 278, children, range by analysis 957–2138 **% Female:** NR **Mean age:** NR **Ethnicity:** NR **Mean maternal education:** NR **At High Risk >50% Children:** Yes	AA or Higher	Cooperative Behavior 16.58 (4.63) PPVT-III 89.1 (NR) Problem Behavior 1.21 (1.47) WJ-Dictation 87.1 (NR) WJ-LWI 92.9 (NR)	**Statistics Extracted:** B **Covariates:** child/family level—age, sex, ethnicity, language, disability, mother-father family, neither birth parent in home, parent literacy, parent education, family income, welfare status, books in home, frequency of reading to child, classroom level—full-day classroom, AP individualizing score, ECERS-R Language, CIS, teacher (a) ratio, (b) experience, (c) DAP beliefs score, (d) ethnicity, (e) salary, parent education, family income, proportion non-minority, proportion language minority, program-level—High Scope curriculum, creative curriculum, teacher salary, proportion non-minority children, parent education, family income, proportion language-minority children
Zill 2006[80]^K^	**Publication:** Report **Design:** Longitudinal **Data Set:** FACES 2000 **Country:** United States **Sample size:** classroom 278, children, range by analysis 674–1729 **% Female:** 50 **Mean age:** NR **Ethnicity:** White-35%, B32%, A1%, H28%, M3%, O1% **Mean maternal education:** NR **At High Risk >50% Children:** Yes	AA or Higher	CAP-One-to-one (NR) CAP-Color naming (NR) Draw-A-Design (NR) PPVT-III (NR) Aggressive 1.49 (1.93) Book Knowledge (NR) Hyperactive 0.97 (1.4) Social Awareness (NR) Social Skills 18.12 (4.28) Withdrawn 2.05 (2.4) WJ-AP (NR) WJ-Dictation (NR) WJ-LWI (NR)	**Statistics Extracted:** B **Covariates**: child/family level—age, gender, ethnicity, disability, parent education, family income, welfare status, language-minority family, mother-father family, neither birth parent in home, parent literacy, books in home, frequency of reading to child, one-year head start graduate, classroom-level—ratio, education, experience, teacher ethnicity, teacher salary, teacher beliefs, CIS, parent education, family income level, proportion language-minority, proportion non-minority, full-day classroom, program-level—parent education, family income, High/scope curriculum, creative curriculum, teacher salary, proportion non-minority children

Abbreviations: NR = Not Reported; C = Caucasian, B = African American, H = Hispanic, A = Asian, M = Mixed, O = Other. For all other acronyms, please refer to S3 File for all child outcomes, and S5 File for all journal, large study, or covariate acronyms.

^a^Descriptives provided reflect characteristics (actual or estimates) of the sample/research design for which data was extracted for the current study and therefore may represent a subsample/analysis of the larger study.

^b^This paper is one of a series of “Meta-Analyses and Systematic Reviews” assessing the relationship between child care quality and children’s outcomes; therefore, uppercase superscript letters below are in reference to various large databases that samples in these papers were drawn from. These letters have been kept consistent across the series of papers for our readers.

^c^Education was operationalized in a number of different ways.

^d^Scale of measurement for the means and standard reported in this table varied across studies (e.g., percentiles, standard scores, raw score). All outcomes used in the current paper are presented in S3 File.

^e^All covariates used in the described sample are listed, but may vary by analyses.

^m^Studies included in the meta analyses.

^**A**^National Center for Early Development and Learning Dataset (NCEDL, 2002, 2004);

^**B**^Head Start Family and Children Experiences Survey (FACES, 2006) Cohort;

^**D**^Cost, Quality and Outcomes Study (CQO, 1993–1994);

^**F**^Georgia Early Childhood Study (GECS, 2002);

^**H**^Early Head Start (EHS, 2001–2003 Cohort);

^**I**^Georgia Pre-K Program (1996–1997);

^**J**^Head Start Family and Children Experiences Survey (FACES, 1997) Cohort;

^**K**^Head Start Family and Children Experiences Survey (FACES, 2000) Cohort;

^**L**^Head Start Family and Children Experiences Survey (FACES, 2003) Cohort;

^**M**^Head Start Family and Children Experiences Survey (FACES, 2009) Cohort;

^**N**^Early Childhood Longitudinal Study (ECLS-B, 2001–2006, Birth Cohort);

^**Q**^National Institute of Child Health and Human Development (NICHD, 1995–1996);

^**S**^8-County Region of North-Central Indiana (Year NR);

^**T**^Otitis Media Study (Year NR);

^**U**^Preschool Curriculum Evaluation Research (PCER, 1999–2003);

^**W**^Mid Atlantic State US (Year 2004–2005; 2005–2006);

^**YA**^More is Four North Carolina Study (2002–2003) Cohort;

^**YB**^More is Four North Carolina Study (2003–2004) Cohort;

^**Z**^Colorado QRIS.

One study was conducted in Canada [68] (covering 4 Atlantic provinces, New Brunswick, Prince Edward Island, Newfoundland and Nova Scotia) and one study used a sample collected across multiple countries [70] (including Finland, Hong Kong, Ireland, Italy, Poland, Thailand, and the United States). The rest of the studies were conducted in the U.S.

Many of the studies had overlapping samples. Nine studies contained samples that were drawn from both the NCEDL’s Multi-State Study and SWEEP study [19,34,48,49,59,66,72,73,81], two used the Head Start FACES 2000 Cohort sample [79,80], three utilized the 2003 Head Start FACES sample [16,49,74], and two studies were based on the 2006 Head Start FACES cohort sample [50,76]. Also, two studies included samples drawn from the NICHD Study of Early Child Care [49,71], and two studies included samples drawn from the Preschool Curriculum Evaluation Research (PCER) Program [49,62]. Furthermore, three studies were based on data from the Georgia Early Childhood Study [49,81,65].

### Description of participants

#### Teachers and programs

The data were collected from the samples of 15 to 887 staff members (median = 242.5) working in 14 to 887 classrooms (median = 257.5) from 16 to 704 child care centers (median = 135). In 14% of the samples used in this study education data were collected from all staff in the classroom and in 66% of the samples education data were collected from one primary caregiver, usually the head staff member (20% of the studies did not report on the staff surveyed). Between 12% and 100% of staff in the study samples reported having a BA or higher (n = 27, median = 55%), or between 13.8 and 16 years of education (n = 7, median = 15.67%). Statistics for the remaining 16 samples were not reported.

#### Children and families

Sample sizes of the included studies ranged from 51 to 3584 children (median = 945), involving 33,175 children overall for non-overlapping samples (i.e., for this purpose we only counted children from each of the databases used by multiple studies once). Between 43% and 56% of children in the study samples are males (median = 50%). Mean age of children across the samples ranges between 36 and 56 months (median = 51 months). The samples contained between 5.5% and 100% of non-Caucasian children (median = 59%). Children from minority ethnic backgrounds were primarily African American, Hispanic and Latino.

Of the 30 non-overlapping samples in this review (i.e., no child counted more than once), 20 authors identified their sample of children as being “at-risk”, 8 indicated that the sample was not “at-risk” and 2 did not provide this type information. The specific details of the SES index used to determine “at-risk” status was rarely reported. Authors indicated that families were poor, came from low-income households, had incomes below a poverty threshold, or were receiving child care subsidies. The percentage of children considered “at-risk” in these samples ranged from 50% and 100% (median = 69%), with the exception of one study (i.e., 38%).

#### Operationalization of staff education

In the studies used in this review, staff education was operationalized in a variety of ways. Detailed information about the way staff education was operationalized in each study included in this review is provided in the Quality Measures column of Table 3. For example, some studies compared staff members with and without an Associate of Arts (AA) or Higher degree [50,52,79,80], a Bachelor of Arts (BA) degree [49,62,65,66], or a BA or Higher degree [16,18,34]. Other studies used the number of years of education, e.g., 1 to16, where a value of 16 would be equivalent to a post graduate degree [54,58–60]. Ordinal scales were also used to capture the level of education measured on 4- to 7-point scales, such as (1) Some college only, (2) AA or 2 year, (3) BA, (4) At least 1 year beyond BA, and (5) MA and above) [46,48,60]. Some studies operationalized staff education in multiple ways and reported separate analyses for each [34,49]. In addition, as ECEC classrooms are staffed with multiple staff, some studies selected only the lead staff [56,57], while other studies collected education data from all staff in the classroom [18,48]. To reflect the different ways in which staff education was operationalized across the studies, we organized the tables for the systematic review into sections based on the type of operationalization that was used in the analysis. Meta-analyses were also conducted separately for each type of operationalization.

### Outcomes

Studies selected for this review provided associations between staff education and the scores of 112 measures of academic competence (e.g., language and math) as well as cognitive, physical and social-emotional development outcomes. All measures are listed in S3 File. The majority of the measures were reported in a single study only. The measures used in the largest number of studies captured receptive language (using the Peabody Picture Vocabulary Test; PPVT) (21 studies, 28 samples) and early math skills (using the Woodcock Johnson-Applied Problems; WJ-AP) (19 studies, 27 samples).

### Systematic review

**Fig 2:**
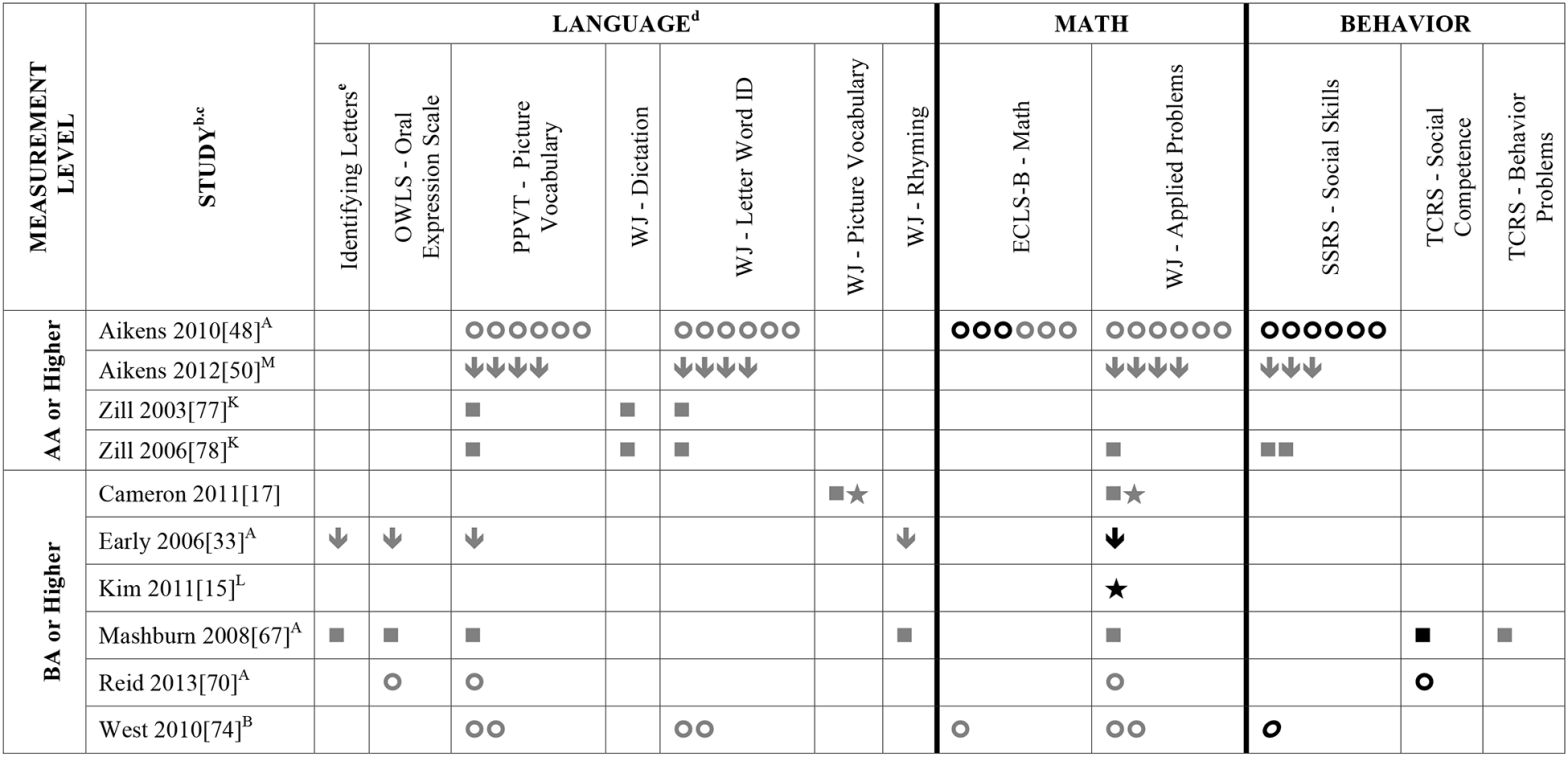
Systematic review of the associations between staff education (AA or Higher; BA or Higher) and child outcomes. ^a^ Abbreviations: AA = Associate’s Degree; BA = Bachelor’s Degree. Symbols bolded are significant and positive, symbols bolded and italicized are significant and negative, and symbols in grey are non-significant. Star = Zero Order Pearson’s Correlation, Unfilled circle = Beta, Filled square = Unstandardized Coefficient, Downward arrow = Effect Size. ^**a**^To improve the readability of this complex table, eleven papers [17,21,55–57,61,63,67,70,71,82] that had an outcome that appeared in only that one paper were omitted from this table. Several analyses from other papers that had idiosyncratic outcomes are also excluded. For a comprehensive display of all of the data for all of the child outcomes see Tables A-H in S4 File. ^**b**^This paper is one of a series of Meta-Analyses and Systematic Reviews assessing the relationship between child care quality and children’s outcomes; therefore, superscript letters below are in reference to various large databases that samples in these papers were drawn from. These letters have been kept consistent across the series for our readers. ^**c**^Samples within papers are described in more detail in Table 3 in the manuscript. ^d^Acronyms for child outcomes are listed in S3 File and for journals, large samples and covariates are in S5 File. ^**e**^Identifying Letters (also refers to as Alphabet Recognition Test, Naming Letters, and Letter-Naming Test). ^**A**^National Center for Early Development and Learning Dataset (NCEDL, 2002, 2004); ^**B**^Head Start Family and Children Experiences Survey (FACES, 2006 Cohort); ^**K**^Head Start Family and Children Experiences Survey (FACES, 2000 Cohort); ^**L**^Head Start Family and Children Experiences Survey (FACES, 2003 Cohort); ^**M**^Head Start Family and Children Experiences Survey (FACES, 2009 Cohort).

**Fig 3:**
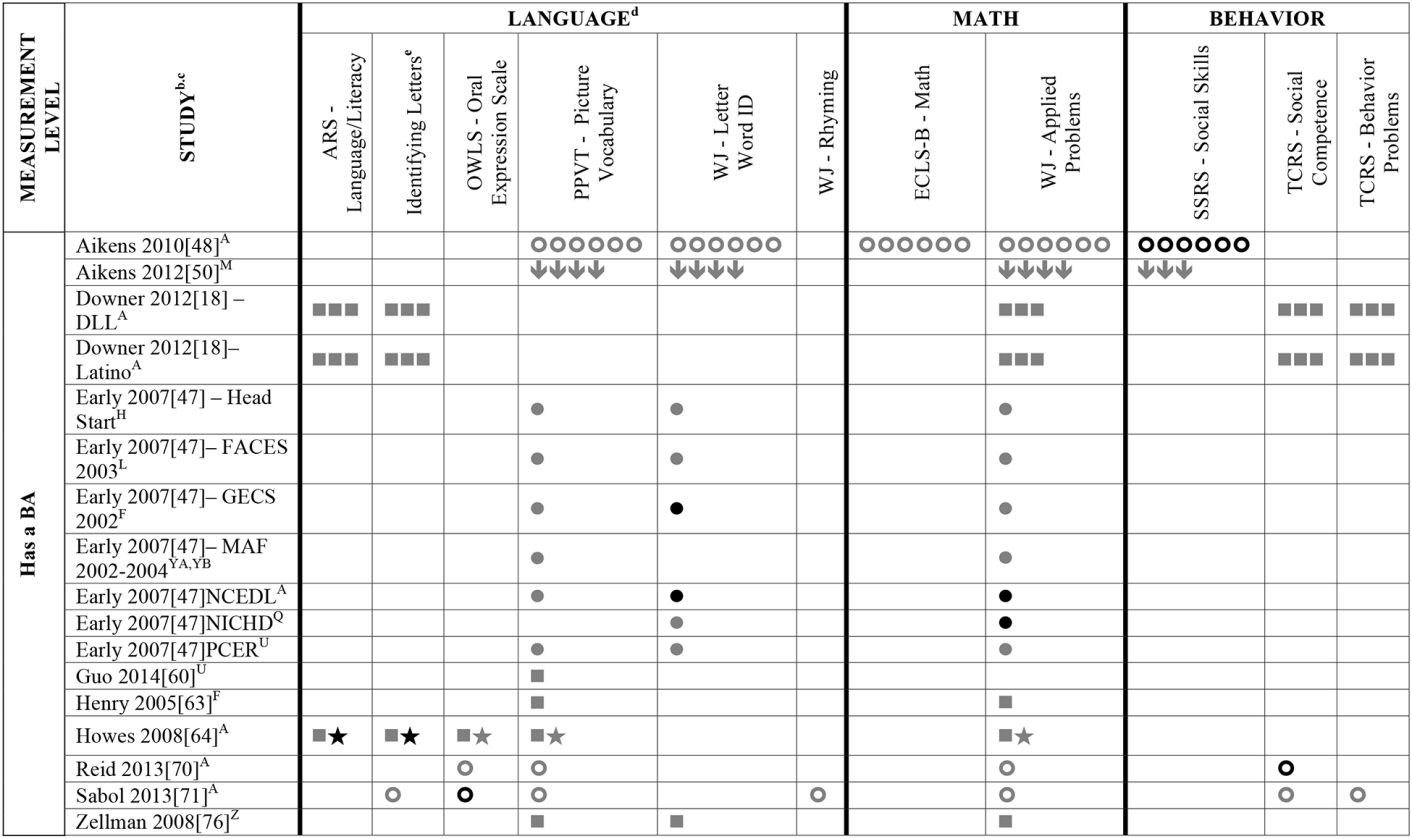
Systematic review of the associations between staff education (Has a BA) and child outcomes. ^a^ Abbreviations: BA = Bachelor’s Degree. Symbols bolded are significant and positive, symbols bolded and italicized are significant and negative, and symbols in grey are non-significant. Star = Zero Order Pearson’s Correlation, Unfilled circle = Beta, Filled square = Unstandardized Coefficient, Downward arrow = Effect Size, Filled circle = F-Ratio. ^**a**^To improve the readability of this complex table, eleven papers [17,21,55–57,61,63,67,70,71,82] that had an outcome that appeared in only that one paper were omitted from this table. Several analyses from other papers that had idiosyncratic outcomes are also excluded. For a comprehensive display of all of the data for all of the child outcomes see Tables A-H in S4 File. ^**b**^This paper is one of a series of Meta-Analyses and Systematic Reviews assessing the relationship between child care quality and children’s outcomes; therefore, superscript letters below are in reference to various large databases that samples in these papers were drawn from. These letters have been kept consistent across the series for our readers. ^**c**^Samples within papers are described in more detail in Table 3 in the manuscript. ^d^Acronyms for child outcomes are listed in S3 File and for journals, large samples and covariates are in S5 File. ^**e**^Identifying Letters (also refers to as Alphabet Recognition Test, Naming Letters, and Letter-Naming Test). ^**A**^National Center for Early Development and Learning Dataset (NCEDL, 2002, 2004); ^**F**^Georgia Early Childhood Study (GECS, 2002); ^**H**^Early Head Start (EHS, 2001–2003 Cohort); ^**L**^Head Start Family and Children Experiences Survey (FACES, 2003 Cohort); ^**M**^Head Start Family and Children Experiences Survey (FACES, 2009 Cohort); ^**Q**^National Institute of Child Health and Human Development (NICHD, 1995–1996); ^**U**^Preschool Curriculum Evaluation Research (PCER, 1999–2003);^**YA**^More is Four North Carolina Study (2002–2003 Cohort); ^**YB**^More is Four North Carolina Study (2003–2004) Cohort; ^**Z**^Colorado QRIS.

**Fig 4:**
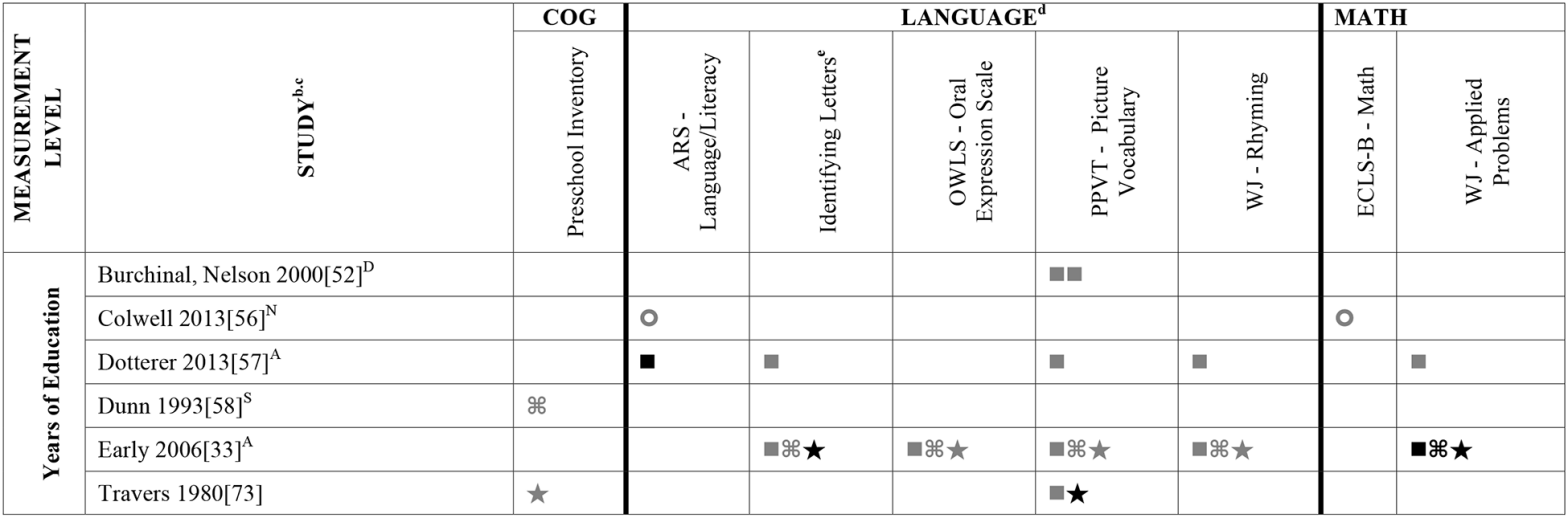
Systematic review of the associations between staff education (Years of Education) and child outcomes. ^a^ Abbreviations: BA = Bachelor’s Degree. Symbols bolded are significant and positive, symbols bolded and italicized are significant and negative, and symbols in grey are non-significant. Star = Zero Order Pearson’s Correlation, Unfilled circle = Beta, Filled square = Unstandardized Coefficient, Key clover = Partial Correlation, Downward arrow = Effect Size. ^**a**^To improve the readability of this complex table, eleven papers [17,21,55–57,61,63,67,70,71,82] that had an outcome that appeared in only that one paper were omitted from this table. Several analyses from other papers that had idiosyncratic outcomes are also excluded. For a comprehensive display of all of the data for all of the child outcomes see Tables A-H in S4 File. ^**b**^This paper is one of a series of Meta-Analyses and Systematic Reviews assessing the relationship between child care quality and children’s outcomes; therefore, superscript letters below are in reference to various large databases that samples in these papers were drawn from. These letters have been kept consistent across the series for our readers. ^**c**^Samples within papers are described in more detail in Table 3 in the manuscript. ^d^Acronyms for child outcomes are listed in S3 File and for journals, large samples and covariates are in S5 File. ^**e**^Identifying Letters (also refers to as Alphabet Recognition Test, Naming Letters, and Letter-Naming Test). ^**A**^National Center for Early Development and Learning Dataset (NCEDL, 2002, 2004); ^**D**^Cost, Quality and Outcomes Study (CQO, 1993–1994); ^**N**^Early Childhood Longitudinal Study (ECLS-B, 2001–2006 Birth Cohort); ^**S**^8-County Region of North-Central Indiana (Year NR).

**Fig 5:**
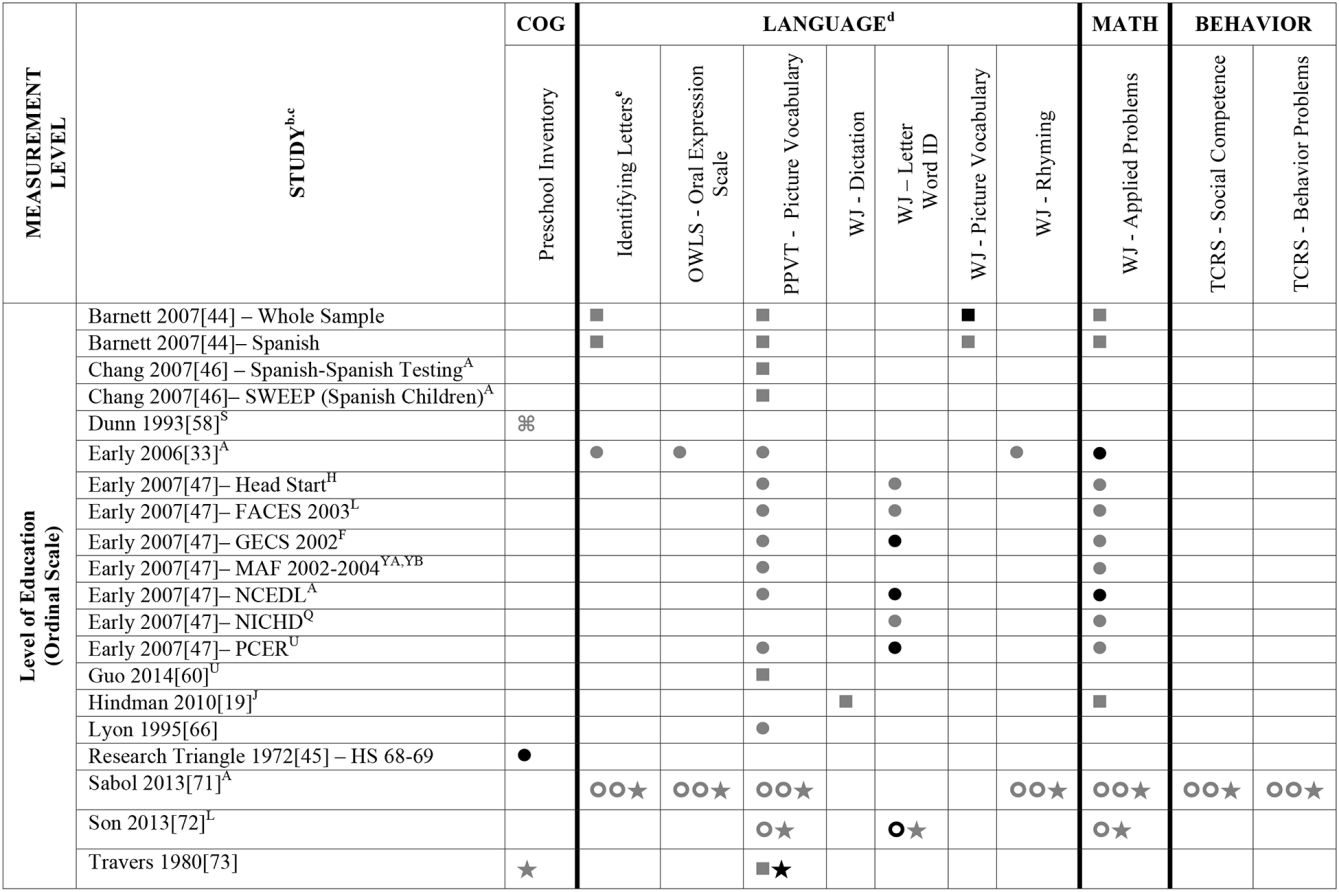
Systematic review of the associations between staff education (Level of Education, Ordinal) and child outcomes. ^a^ Abbreviations: BA = Bachelor’s Degree. Symbols bolded are significant and positive, symbols bolded and italicized are significant and negative, and symbols in grey are non-significant. Star = Zero Order Pearson’s Correlation, Unfilled circle = Beta, Filled square = Unstandardized Coefficient, Key clover = Partial Correlation, Downward arrow = Effect Size, Filled circle = F-Ratio. ^**a**^To improve the readability of this complex table, eleven papers[17,21,55–57,61,63,67,70,71,82] that had an outcome that appeared in only that one paper were omitted from this table. Several analyses from other papers that had idiosyncratic outcomes are also excluded. For a comprehensive display of all of the data for all of the child outcomes see Tables A-H in S4 File. ^**b**^This paper is one of a series of Meta-Analyses and Systematic Reviews assessing the relationship between child care quality and children’s outcomes; therefore, superscript letters below are in reference to various large databases that samples in these papers were drawn from. These letters have been kept consistent across the series for our readers. ^**c**^Samples within papers are described in more detail in Table 3 in the manuscript. ^d^Acronyms for child outcomes are listed in S3 File and for journals, large samples and covariates are in S5 File. ^**e**^Identifying Letters (also refers to as Alphabet Recognition Test, Naming Letters, and Letter-Naming Test). ^**A**^National Center for Early Development and Learning Dataset (NCEDL, 2002, 2004); ^**F**^Georgia Early Childhood Study (GECS, 2002); ^**H**^Early Head Start (EHS, 2001–2003 Cohort); ^**J**^Head Start Family and Children Experiences Survey (FACES, 1997 Cohort); ^**L**^Head Start Family and Children Experiences Survey (FACES, 2003 Cohort); ^**Q**^National Institute of Child Health and Human Development (NICHD, 1995–1996); ^**S**^8-County Region of North-Central Indiana (Year NR); ^**U**^Preschool Curriculum Evaluation Research (PCER, 1999–2003);^**YA**^More is Four North Carolina Study (2002–2003 Cohort); ^**YB**^More is Four North Carolina Study (2003–2004) Cohort.

Data summarizing all of the findings from the 39 eligible studies (50 samples of data) are presented Tables A-H in S4 File (A snapshot summary of just those outcomes analyzed in three or more samples is also provided in Figs 2 to 5). The first column in all these tables and figures indicates the type of operationalization of staff education used in the reviewed studies. The results of statistical analyses are presented in these tables using a variety of symbols indicating the type of analysis and its statistical significance. To ensure the comprehensiveness of our review, all models tested in each of the papers are included. Each symbol represents a unique model. This allows the reader to assess how many effects were significant given the total number of different models/analyses authors reported in each of these studies. For example, in Fig 2, Aikens (2010) [50], conducted linear regressions for 6 different models, reporting Beta scores for each.

To facilitate interpretation, these tables are grouped based on the types of outcomes reported in the study. Across all eligible studies, 477 distinct statistical analyses quantifying the association between staff education and child outcomes were reported. These statistical analyses included 112 unique child outcome measures associated with the different ways of operationalizing staff education described above (see S3 File).

#### Approach to learning outcomes

Two studies [61,78] reported an association between staff education and approach to learning outcomes. Zellman (2008) [78] used five subscales of Child Behavior Inventory measure and Epstein (1993) [61] used the Initiative subscale of Child Observation Record measure. Both studies showed a non-significant association between this type of child outcomes and staff education.

#### Cognitive outcomes

Fifteen studies reported an association between staff education and cognitive outcomes with 17 different measures (listed in S3 File). Most of these measures were used in a single study with the exception of Intelligence subscale of the Child behavior Inventory (2 studies), the Color Naming test (2 studies), and the Preschool Inventory (3 studies). The majority of these studies found no association between staff education and this type of child outcome. Only 3 studies [17,47,71] reported significant relationships. However, the results of these studies are mixed: while two studies [47,71] show a positive relationship between staff education and school readiness and intelligence, the study by Mashburn (2004) [17] found a negative relationship between staff education and child academic skills and school readiness.

#### Language outcomes

The associations between staff education and child language outcomes were reported for 43 samples drawn from all 39 studies included in this systematic review. The results for 35 different measures of language development were reported. These measures were used to evaluate language development as a whole (e.g., Language subscales from Child Observation Record, Academic Rating Scale, and DIAL-R) or some aspects of language development, such as letter recognition, vocabulary, phonological awareness, and rhyming. Most of these measures were used in a single study. Only 8 measures were used in 2 or more studies. The Peabody Picture Vocabulary Test (PPVT) was reported in the largest number of studies [31].

The vast majority of the results in these studies showed no significant association between staff education and child language outcomes. A small number of significant results showed a positive relationship between the two types of indicators. However, these significant results came from the studies that report multiple child language outcomes and mixed findings across the outcomes (e.g., Howes 2008, Sabol 2013) [66,73].

#### Mathematics outcomes

Association between staff education and 7 different math outcomes was reported for 32 samples in 23 studies. The WJ Applied Problems measure of child competency in mathematics is the only outcome that was reported in a large number of studies (n = 19). The rest of the math outcomes were reported in 1to 3 studies. Most of the results reported across these studies suggest a lack of association between staff education and math outcomes. A few significant results indicated a positive association and are reported in two papers only [34,49].

#### Physical health and development outcomes

Two studies reported the relationship between staff education and child physical development outcomes measured with three different indicators. None of the results in these studies showed a significant association.

#### Social-emotional: Positive behavior outcomes

The results of statistical analyses investigating the relationship between staff education and child positive behavior were reported for 25 different outcomes in 9 studies. Most of these outcomes were used in a single study. Each study involved multiple indicators of positive behavior and multiple ways of operationalizing staff education. Two outcomes (Social Skills Rating System and Teacher Child Rating Scale) were reported in 4 studies each.

The studies included in this systematic review show mixed results about the association between staff education and positive behavior. For most indicators reported in a single study the results are not significant. However, for the two indicators reported in multiple studies (Social Skills Rating System and Teacher Child Rating Scale), two out of four studies showed a significant positive association between staff education and positive behavior.

#### Social-emotional: Problem behavior outcomes

Problem behavior was measured in 17 samples used in 15 studies with 15 different outcome variables. With exception of Behavior Problems subscale from the Teacher Child Rating Scale that was used in three studies, all other outcomes were used in a single study. Most studies reported a single problem behavior outcome.

The majority of the reported results showed no significant association between staff education and problem behavior outcomes. A few significant results show positive relationship between higher levels of staff education and decreases in child behavior problems during the school year [47,71,80].

Overall, the vast majority of the results reported in the 39 studies (50 samples) we reviewed suggest small or no associations between staff education and children’s academic competence (e.g., language, mathematics), as well as cognitive, physical and social-emotional outcomes. The characteristics of studies that reported significant associations were compared with studies that did not. For example, we qualitatively explored whether studies conducted more recently showed more/fewer associations between education and child outcomes than older studies. We adopted a similar strategy to exploring study design (e.g., cross-sectional vs. longitudinal), operationalization of staff education (e.g., years of education vs. degrees) and whether authors reported statistics that did or did not account for covariates (i.e., beta coefficients from regressions vs. Pearson correlations and F-ratios). The small number of significant effects that were found were reported across studies with these different characteristics. This suggests that these characteristics do not explain why a very small number of studies that reported significant effect did so, while the others did not.

### Meta-analyses

**Fig 6:**
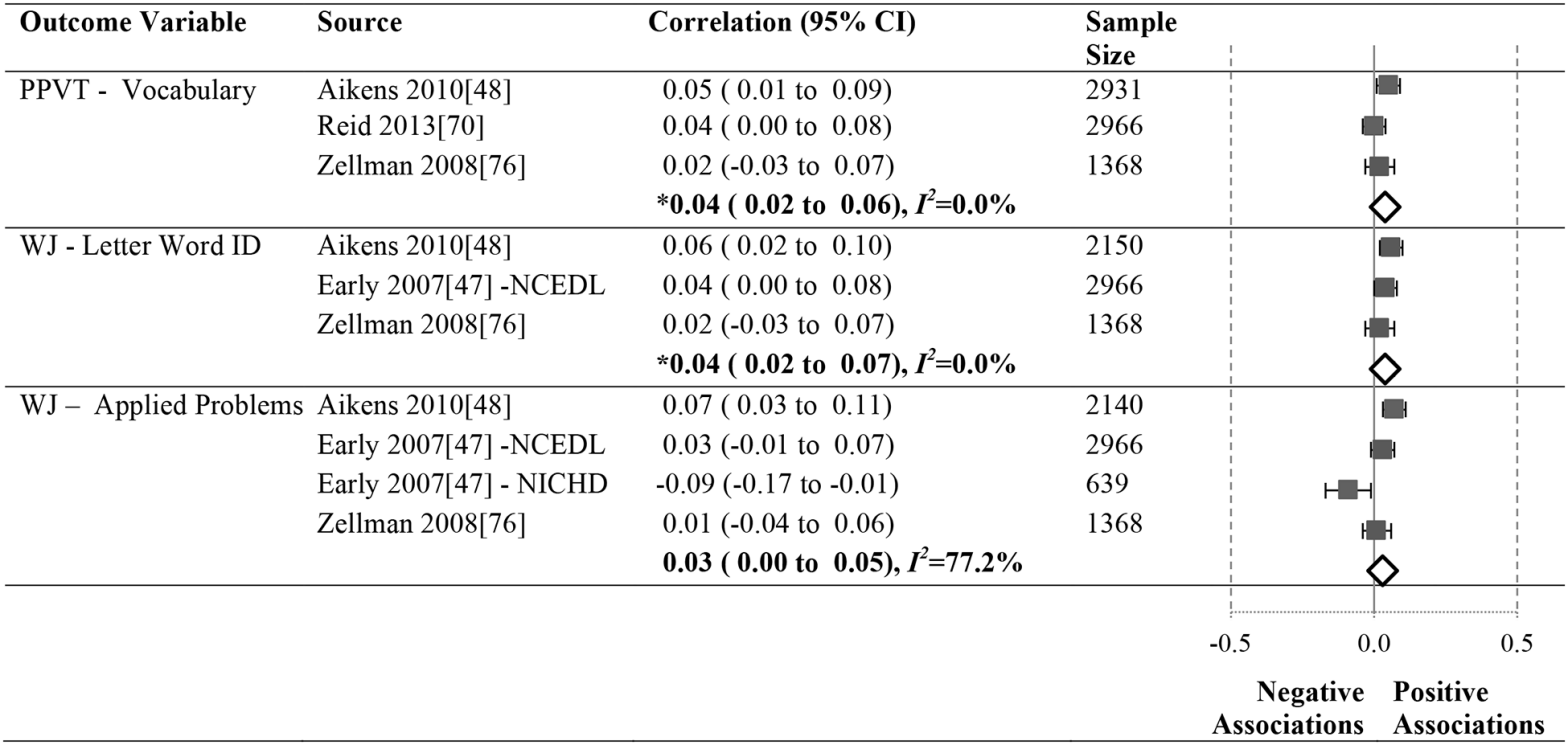
Meta-analysis results for the associations between staff education measured as a dichotomy, having a BA or not, and child outcomes. Significant findings are noted with asterisks.

**Fig 7:**
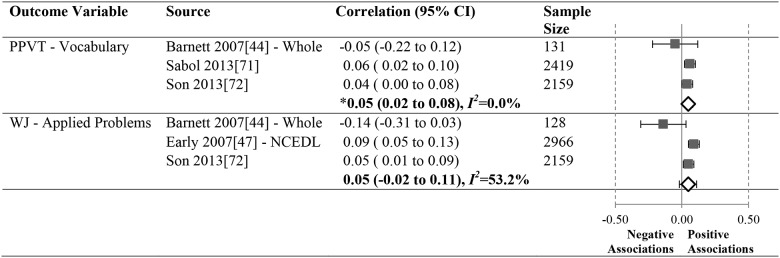
Meta-analysis results for the association between staff education measured as level of education (ordinal) and child outcomes. Significant findings are noted with asterisks.

We identified that 3 outcomes were used in studies with the similar operationalization of education fulfilling the criteria for meta-analyses. These were: one math outcome (WJ Applied Problems) and two language outcomes (PPVT and WJ Letter Word ID). Meta-analyses in these studies were based on three to four studies. The results of meta-analyses relating teachers’ BA degree with these outcomes are presented in Fig 6 and those relating teachers’ level of education to these outcomes are presented in Fig 7.

The meta-analysis identified a small positive relationship between the PPVT and staff education (pooled *r* = 0.04, 95% confidence interval 0.02 to 0.06, *I*^*2*^ = 0%). Similarly, we identified a weak positive association between this WJ-Letter Word ID outcome and staff education (pooled *r* = 0.04, 95% confidence interval 0.02 to 0.07, *I*^*2*^ = 0%). However, pooled results for the mathematics outcome (WJ Applied Problems, based on 4 studies, 7113 children) showed no association between these variables (pooled *r* = 0.03, 95% confidence interval 0.00 to 0.05, *I*^*2*^ = 77.2%).

Two meta-analyses were conducted to evaluate associations between level of staff education measured by ordinal categories, such as having high school diploma, AA, BA, or a Graduate degree [49] with child outcomes (see Fig 7). Pooled results for the PPVT (based on 3 studies, 4709 children) showed a significant positive association between these two variables (pooled *r* = 0.05, 95% confidence interval 0.02 to 0.08, *I*^*2*^ = 0.0%). The analysis of WJ-Applied Problems was based on three studies (5253 children). The results showed no significant association between this outcome and staff education (pooled *r* = 0.05, 95% confidence interval—0.02 to 0.07, *I*^*2*^ = 53.2%).

## Discussion

The goal of this comprehensive systematic review was to identify associations between staff education and academic competence (e.g., language, mathematics), as well as cognitive, physical and social-emotional outcomes of pre-school children in ECEC settings. We found that some studies reported weak positive associations between staff education and certain outcomes whereas the majority of studies reported no association. Marked heterogeneity in assessment of education and outcomes was identified and is discussed below. A few studies that defined education and reported outcomes in a similar manner were meta-analyzed and revealed weak positive associations between staff education and receptive language and letter identification. It is important to note that since much of the variability in child outcomes is driven by child and family factors, even small effects associated with ECEC programs, particularly when family factors are accounted for, may be meaningful. This is especially true when language is the outcome as it has been shown that effects on language outcome can have long lasting effects [83] that spillover into multiple domains of a child’s life including social, emotional and cognitive outcomes [84,85]. The finding that staff education is associated only with language outcomes may reflect recent emphasis on language in literacy in ECEC staff training programs or it may reflect the predispositions of the individuals who are drawn to careers in ECEC settings, who may be more oriented towards language than math. However, the results from our meta-analysis represent a small minority of selected studies and should be viewed with caution and as hypothesis generating. The lack of associations with other outcomes (e.g., classroom management styles and children’s behavioral outcomes) is unexpected and requires further research to identify avenues for improvement.

We explored whether including all staff in the room was associated with a different pattern of results than including the education of the lead staff members only. A substantially higher proportion of studies that used only the lead teacher’s education in the analyses reported one or more significant effect, relative to studies that used aggregated education level of all staff in a classroom. This is surprising given that exploring the education of head-teachers only provides a partial picture of the education of staff in a given classroom. One possible explanation is that the lead teachers’ education is more important in terms of driving children’s experiences in the classroom and therefore their outcomes than the education levels of the other staff. Another, is that the methods researchers use to aggregate the data when they collected education levels from all staff (e.g., averaging or weighing some staff more than others) result in a classroom level index that did not reflect children’s experiences in the classrooms. Unfortunately, reporting of the method of aggregation is highly inconsistent across studies and did not allow us to explore this issue. It is important to note, however, that only 7 studies assessed the education of all staff in the room while 32 assessed the lead teachers’ education only. Given these low numbers, the above analysis needs to be interpreted with caution.

We also explored whether the pattern of results was different in studies in which the sample was considered “at-risk” (see Table 3). To do this we compared the number of samples that reported one or more significant effects in studies in which the sample was not “at-risk” (as described by the authors) vs. studies where the sample consisted of 50% or more children considered “at-risk” (based on poverty). Overall, the proportion of samples that reported significant results was similar regardless of the sample “at-risk” status. This suggests that the differential susceptibility hypothesis is not at play for staff education in the literature included in this review. However, this qualitative analysis, based on a small number of samples must be interpreted with caution.

### Heterogeneity in research on associations between staff education and child outcomes

The body of research we reviewed is very heterogeneous in terms of the operationalization of staff education, reported statistics, covariates used in the analyses, and child outcome measures used. Our review identified 112 distinct outcome measures that spanned a wide range of abilities. These were collected through direct assessment of children and through surveys of parents and ECEC staff. Thus, there was substantial heterogeneity in what was measured and how the information was gathered. Studies relied on different analyses and therefore reported different statistics. Some studies reported zero-order correlations, others reported only the results of regression analyses. Where possible we converted statistics to maximize the number of studies that could be meta-analyzed but the information needed to do this was not always available in the papers. When researchers conducted regressions the number and nature of covariates they included varied. Finally, staff education was operationalized in a variety of different ways across the reviewed studies (number of years of education, attainment of a particular level/degree, levels of education measured with ordinal categories).

Reporting of study methodology and results is inconsistent and at times incomplete and limited our ability to integrate across papers. For example, some of the studies did not report the demographics of the sample making it hard to understand the generalizability of the findings [52,61]. Other studies did not report the descriptive statistics for quality indicators and/or child outcomes [46,57,61]. Clearly, greater consistency in methodologies and reporting is needed to facilitate integration across studies in the future.

We dealt with this substantial heterogeneity by being inclusive in our systematic review while taking a conservative approach to ensure that the studies we meta-analyzed were sufficiently similar to one another. Specifically, for the meta-analyses we selected outcomes that were used in three or more papers, which meant that the outcome measures that were meta-analyzed tended to be strong psychometrically. We did not combine statistics that did and did not include covariates and we reduced heterogeneity in exposure to ECEC programs by only including studies that ensured that children had had at least some exposure to their ECEC program. The downside of this approach is that it limited the number of papers that could be meta-analyzed. The upside is that we reduced the heterogeneity of what we meta-analyzed thereby increasing our confidence that the studies we meta-analyzed were sufficiently similar to be combined.

### Methodological limitations of research on associations between staff education and child outcomes

A major methodological issue in this area of research is the reliance on observational/correlational studies based on samples that are not randomly selected. Researchers and policy makers need to work together to look for opportunities to study the effects of staff education in naturally occurring settings such as changes to regulation with regard to staff education. Another issue is that children and staff are nested in classrooms that are nested in centers. Yet, the lack of independence between units of analyses is often not accounted for in the statistics researchers in this area use. In addition, there is often a mismatch between the unit of analysis of staff education (generally measured at the staff or classroom level) and child outcomes (which are measured at the child level). With a few exceptions, the studies included in this review were based on U.S. samples. Future research needs to expand on the status of this important aspect of ECEC quality in other cultures.

### Conceptual explanations of the weak associations between staff education and child outcomes

Several conceptual factors may explain the limited association between staff education and child outcomes that we identified. Other staff characteristics such as the area of specialization, years of experience, professional development opportunities, and knowledge of child development may need to be considered simultaneously. Having a particular level of education may not result in the assumed knowledge that would help staff support children’s development. This may be because of a potential disconnect between the content covered in formal education programs and what makes for good quality experiences for children. Alternatively, even if staff are taught (and retain what they are taught) about best practices for supporting child development, they might not implement what they learn. For example, researchers have found that educators’ practices are driven by previous beliefs, knowledge, past practices [86] as well as their own childhood experiences [87], rather than best practices. A focus on what staff *do* may be more productive than focusing on their education as a proxy of their behavior. There is some support for this idea from the parenting literature. Findings from the Effective Provision of Preschool Education Project based on a short interview asking parents about what they do with their children identified that, “While other family factors such as parents' education and SES are also important, the extent of home learning activities exerts a greater and independent influence on educational attainment” (p. 106) [88]. Perhaps this is also true of staff in the ECEC sector. The increasing focus in the literature on staff-child interactions is consistent with this emphasis. However, our recent review of staff/child interactions (the Classroom Assessment Scoring System) [89] also identified a few associations with child outcomes [90]. Finally, the differential susceptibility hypothesis suggests that some children are more sensitive to variability in the quality of their environments [91]. Thus, having better-educated staff may be important for some (e.g., at-risk) children. While we did not find such a pattern in this review, due to the limited number of studies available for meta-analysis, we were not able to test this statistically. Despite adding to the complexity of an already highly complex research area, these conceptual issues cannot be ignored for the field to move forward.

### Limitations of the current study

One of the main limitations of the current study is the methodological limitations of the literature we covered which were described above. In addition, the small number and heterogeneous nature of the studies included in this paper severely limited our ability to meta-analyze across papers. Further, due to the small number of studies included in our meta-analyses, the effects of possible moderators on the relationship between staff education and child outcomes could not be examined. Moderating variables (e.g., does education matter when staff/child ratios are poorer?) should be explored once more scientific evidence is collected and presented in more homogeneous way.

## Conclusion

Results from our systematic review were hampered by heterogeneity in the definition of staff education, variability in whether all or only some staff’s education was measured, as well as variability in the child outcomes that were collected. However, overall the qualitative summary indicates that associations between staff education and childhood outcomes are, at best, non-existent to very borderline positive. In our meta-analysis of more homogeneous studies, we identified certain positive, albeit very weak, associations between staff education and children’s language outcomes (specifically, vocabulary and letter word identification) while no significant associations were found with a mathematics outcome (WJ Applied Problems). Nonetheless, by compiling the existing literature in a systematic way, this study has highlighted a number of important methodological issues that need to be addressed in future research. While the meta-analyses revealed a few associations, the lack of associations is meaningful as it draws researchers, policy makers and other stakeholders’ attention to other avenues for improving quality in ECEC settings. These include examining practices in settings that train early childhood educators and the professional development opportunities available in this area, as well as assessing how staff interact with children rather than using structural variables such as education as measures of ECEC program quality.

## Supporting information

S1 File Search syntax for staff education. (PDF)

S2 File Formulas for staff education. (PDF)

S3 File Child outcomes for staff education. (PDF)

S4 File Systematic review tables for staff education. (PDF)

S5 File Acronyms for staff education. (PDF)

S6 File Database for staff education. (ZIP)

S7 File Prisma checklist. (PDF)
