# Supplementary material for: Education of staff in preschool aged classrooms in child care centers and child outcomes: A meta-analysis and systematic review
Source: PLoS One. 2017 Aug 30;12(8):e0183673. doi: 10.1371/journal.pone.0183673 (PMC5576714; doi:10.1371/journal.pone.0183673)
Supplement: S2 File — (PDF) [file pone.0183673.s002.pdf]

Education of Staff in Preschool Aged Classrooms in Child Care Centers and Child Outcomes: A Meta-Analysis and Systematic Review

Supplemental Information 2

Formulas for Converting Statistics to r for Meta Analyses

Conversion to *t*, *d* or *r* through *t*-statistic

$$t = \frac{B}{SE(B)}$$

$$d = \frac{2t}{\sqrt{n-1}}$$

$$r = \frac{d}{\sqrt{d^2 + 4}}$$

where *B* is an unstandardized regression coefficient, *SE(B)* is its standard error, *d* is its standardized mean difference, *r* is the correlation effect size, and *n* is sample size.
