# Supplementary material for: Education of staff in preschool aged classrooms in child care centers and child outcomes: A meta-analysis and systematic review
Source: PLoS One. 2017 Aug 30;12(8):e0183673. doi: 10.1371/journal.pone.0183673 (PMC5576714; doi:10.1371/journal.pone.0183673)
Supplement: S3 File — (PDF) [file pone.0183673.s003.pdf]

Education of Staff in Preschool Aged Classrooms in Child Care Centers and Child Outcomes: A Meta-Analysis and Systematic Review

Supplemental Information 3

Staff Education Quality Indicator: List of Child Outcome Measures

Outcome Category	Number of Measures	Child Outcome Variable	Number of Studies	Number of Samples
Approach	n=6	Child Behavior Inventory - Creativity (CBI)	1	1
		Child Behavior Inventory - Dependence (CBI)	1	1
		Child Behavior Inventory - Distractibility (CBI)	1	1
		Child Behavior Inventory - Independent (CBI)	1	1
		Child Behavior Inventory - Task Orientation (CBI)	1	1
		Child Observation Record - Initiative (COR)	1	1
Cognitive	n-17	Backward Digit Span	1	1
		Bayley Scales of Infant Development: Mental Development Index (BSID)	1	1
		Bracken Basics Concepts Scale	1	1
		Child Behavior Inventory - Intelligence (CBI)	2	2
		Child Behavior Scale(CBS)	0	0
		Cognitive (author created)	1	1
		DIAL-R - Concepts (DIAL-R)	1	1
		Faces Social Awareness Task	1	1
		Identifying Colors (Color Naming)	2	2
		Intellectual Ability	1	1
		Preschool Inventory (PSI)	3	3
		Retention Rates	1	1
		Stanford 9 - Science	1	1
		Stanford 9 - Social Science	1	1
		Stanford Binet	1	2
		Student Rating Form - Academic Skills Factor	1	1
		Student Rating Form - Kindergarten Readiness	1	1
		Wechsler Preschool Primary Scale of Intelligence (WPPSI)	1	1
Combo	n=4	Child Observation Record - Representation (COR)	1	1
		Child Observation Record - Total (COR)	1	1
		Combination of all Child Outcomes	1	1
		DIAL-R - Total (DIAL-R)	1	1
Language	n=35	Academic Rating Scale: Language & Literacy	4	5
		Child Observation Record - Language (COR)	1	1
		DIAL-R - Language (DIAL-R)	1	1
		Emergent Literature Composite	1	1
		Expressive One Word Picture Vocabulary Test (EOWPVT, English)	1	1
		Expressive One Word Picture Vocabulary Test (EOWPVT, Spanish)		
		Identifying Letters (Alphabet Recognition, Naming Letters, Letter-Naming Test)	7	9
		Identifying Letters (Spanish, Alphabet Recognition)		
		Language (author created)	1	1
		Language (PPVT + WJ Picture Vocabulary)	1	1
		Oral & Written Language Scales - Oral Expression Scale (OWLS)	5	5
		Oral & Written Language Scales - Total Scale (OWLS)	1	1
		Peabody Picture Vocabulary Test 4 (PPVT-4)	21	28
		Peabody Picture Vocabulary Test III (PPVT-III)		
		Peabody Picture Vocabulary Test Revised (PPVT-R)		
		Peabody Picture Vocabulary Test (TVIP)		
		Phoneme Deletion - English	1	2
		Phoneme Deletion - Spanish		
		Phonological Awareness and Literacy Screening-Pre-Kindergarten - Emergent Literacy Composite (PALS Pre-K)	1	1
		Pre-CTOPP - Blending Sounds (Pre-CTOPP)	1	1
		Pre-CTOPP - Elision (Pre-CTOPP)	1	1
		Pre-CTOPP - Print Awareness (Pre-CTOPP)	1	1
		Pre-CTOPP - Receptive Vocabulary (Pre-CTOPP)	1	1
		PreLAS 2000 (PreLas 2000)	1	1

Education of Staff in Preschool Aged Classrooms in Child Care Centers and Child Outcomes: A Meta-Analysis and Systematic Review

Staff Education Quality Indicator: List of Child Outcome Measures

Outcome Category	Number of Measures	Child Outcome Variable	Number of Studies	Number of Samples
		Preschool Language Scales - 3 (PLS-3)	1	1
		Reynell Developmental Language Scales - Expressive Language (RDLS)	1	1
		Reynell Developmental Language Scales - Language Comprehension (RDLS)	1	1
		Rhyme Recognition - English<br>Rhyme Recognition - Spanish	1	2
		SICD-Expressive Communication Age (SICD-ECA)	1	1
		SICD-Receptive Communication Age (SICD-RCA)	1	1
		Stanford 9 - Language Arts	1	1
		Story and Print Concepts – Book Knowledge	1	1
		Story and Print Concepts - Total Score	1	1
		Test of Preschool Early Literacy - Phonological Awareness + Print Knowledge) (TOPEL)	1	1
		Student Rating Form - Communication Skills Factor	1	1
		Woodcock Johnson - Academic Knowledge (WJ-III-AK)	1	1
		Woodcock Johnson- Dictation (WJ-III-D)<br>Woodcock Johnson- Dictation (WJ-R-D)<br>Woodcock Johnson- Dictation (Spanish, WM-III-D)	3	3
		Woodcock Johnson- Letter Word Identification (WJ-III LWI)<br>Woodcock Johnson- Letter Word Identification (WJ-R LWI)<br>Woodcock Johnson- Letter Word Identification (WM LWI, Spanish)	8	13
		Woodcock Johnson- Passage Comprehension (WJ-III-PC)	1	1
		Woodcock Johnson- Picture Vocabulary (WJ-III-PV)<br>Woodcock Johnson- Picture Vocabulary (WJ-R-PV)<br>Woodcock Johnson- Picture Vocabulary (WM—R-PV, Spanish)	2	3
		Woodcock Johnson- Rhyming (WJ-III-R), Sound Awareness (WJ-III-SA)	4	4
Math	n=7	Child Observation Record - Logic & Math (COR)	1	1
		Counting Task	1	1
		Early Childhood Longitudinal Study-Birth Cohort - Math (ECLS-B)	3	3
		Identifying Numbers (Naming Numbers)	2	2
		Stanford 9 - Math	1	1
		Test of Early Mathematics Ability-3 (TEMA-3)	1	1
		Woodcock Johnson- Applied Problems (WJ-III -AP)<br>Woodcock Johnson- Applied Problems (WJ-R-AP)<br>Woodcock Johnson- Applied Problems (WM-R-AP, Spanish)	19	27
Physical	n=3	Child Observation Record - Music & Movement (COR)	1	1
		Design Copying	1	1
		DIAL-R - Motor (DIAL-R)	1	1
Positive Behavior	n=25	Child Behavior Inventory - Considerateness (CBI)	1	1
		Child Behavior Inventory - Sociability (CBI)	1	1
		Child Observation Record - Social Relations (COR)	1	1
		Comply with Parents	1	1
		Comply with Requests	1	1
		Cooperative Behavior	1	1
		Early Childhood Longitudinal Study-Birth Cohort - Approaches to Learning (ECLS-K)	1	1
		Early Childhood Longitudinal Study-Birth Cohort – Attention and Concentration (ECLS-B) (parent and teacher version)	1	1
		Early Childhood Longitudinal Study-Birth Cohort – Emotional and Behavioral Regulation (ECLS-B) (parent and teacher version)	1	1
		Early Childhood Longitudinal Study-Kindergarten Cohort - Prosocial (ECLS-K) (parent and teacher version))	1	1
		Executive Functioning Pencil Tapping Task	2	2
		General Compliance	1	1
		Gumpgookies (GUMP)	1	1

Education of Staff in Preschool Aged Classrooms in Child Care Centers and Child Outcomes: A Meta-Analysis and Systematic Review

Staff Education Quality Indicator: List of Child Outcome Measures

Outcome Category	Number of Measures	Child Outcome Variable	Number of Studies	Number of Samples
		Headtotoes Self-Regulation	1	1
		Positive Behavior (author created) (Teacher)	1	1
		Preschool Learning Behavior Scale - Positive Behavior	1	1
		Social - Cognitive Ability	1	1
		Social - Competence with Stranger	1	1
		Social - Competence with Visitor	1	1
		Social Skills (SS, author created)	1	1
		Social Skills and Behavior Problems Scale - Social Competence (SSPBS)	1	1
		Social Skills and Positive Approach to Learning (Parent)	1	1
		Social Skills Rating System - Social Skills (SSRS-SS) (parent and teacher version)	4	4
		Student-Teacher Relationship Scale - Closeness (STRS)	1	1
		Teacher Child Rating Scale - Social Competence (TCRS)	4	5
Problem Behavior	n=15	Behavior Problems (PB, author created)	3	3
		Behavior Problem Scale (PBS)	1	2
		Caregiver Teacher Report Form/25 (CTRF/25)	1	1
		Child Behavior Inventory - Apathy (CBI)	1	1
		Child Behavior Inventory - Hostility (CBI)	1	1
		FACES - Aggressive Behavior) (parent and teacher version)	1	1
		FACES - Hyperactive Behavior) (parent and teacher version)	1	1
		FACES - Withdrawn Behavior) (parent and teacher version)	1	1
		Feeling of Inadequacy Scale	1	2
		Motivational Problem Scale	1	2
		Problem Behavior Inventory -Problem Behavior (PBI)	0	0
		Problem Behavior Questionnaire - Hyperactivity-Distractibility (Parent) (PBQ)	1	1
		Preschool Behavior Scale - Hyperactive (Teacher) (PBS)	1	1
		Social Skills and Behavior Problems Scale - Behavior Problems (SSBS)	1	1
		Social Skills Rating System - Problem Behaviors (SSRS)	1	1
		Student Teacher Rating Scale – Conflict (STRS)	1	1
		Teacher Child Rating Scale - Behavior Problems (TCRS)	3	4
**Total**	**n=112**			
