# Supplementary material for: Education of staff in preschool aged classrooms in child care centers and child outcomes: A meta-analysis and systematic review
Source: PLoS One. 2017 Aug 30;12(8):e0183673. doi: 10.1371/journal.pone.0183673 (PMC5576714; doi:10.1371/journal.pone.0183673)
Supplement: S4 File — (PDF) [file pone.0183673.s004.pdf]

Supplemental Information 4
Systematic Review Results: All Outcomes

Table A. Staff Education Predicting all Approach, Combination, and Physical Outcomes

**Staff Education Measurement Level**	**STUDY[a, b]**	**APPROACH[c]**						**COMBO**				**PHYSICAL**		
		CBI - Creativity	CBI - Dependence	CBI - Distractibility	CBI - Independence	CBI - Task Orientation	COR- Initiative	COR - Representation	COR - Total	Composite (all child outcomes)	DIAL-R - Total	COR – Music & Movement	Design Copying	DIAL-R - Motor
**College or Higher**	NICHD 1999[1][Q]									●				
**Associate's Degree or Higher**	Zill 2006[2][K]												■	
**Has a Bachelor's Degree**	Zellman 2008[3][Z]	■	■	■	■	■								
**Education - Years**	Epstein 1993[4]						★	★	★		★	★		★

Legend for Table

**Significant and Positive**	**Significant and Negative**	**Nonsignificant**	**Statistic**
★	★	★	**r - Zero Order Pearson's Correlation**
■	■	■	**B (Unstandardized Coefficient)**
●	●	●	**F-Ratio**

[a]This paper is one of a series of Meta-Analyses and Systematic Reviews assessing the relationship between child care quality and children's outcomes; therefore, superscript letters below are in reference to various large databases that samples in these papers were drawn from. These letters have been kept consistent across the series for our readers.
[b]Samples within papers are described in more detail in Table 2 in the manuscript.
[c]Acronyms for child outcomes are listed in Supplemental Information 3.
[Q]National Institute of Child Health and Human Development (NICHD, 1995-1996); [K]Head Start Family and Children Experiences Survey (FACES, 2000) Cohort; [Z]Colorado QRIS.

Supplemental Information 4
Systematic Review Results: All Outcomes

Table B. Staff Education Predicting all Cognitive Outcomes

**Staff Education Measurement Level**	**STUDY[a, b]**	**COGNITIVE[c]**																	
		Backward Digit Span	Bayley – Mental Development	BBCS – School Readiness	CBI - Intelligence	Cognitive (author created)	DIAL-R - Concepts	FACES - Social Awareness	Identifying Color[d]	Intellectual Ability	Preschool Inventory	Retention Rates	Stanford Binet	Stanford 9 - Science	Stanford 9 – Social Science	SRF - Academic Skills	SRF - K Readiness	Wechsler Intelligence
**College or Higher**	Burchinal, Roberts 2000[5][T]		★															
	Henry, Gordon 2003[6][I]											■		■	■			
	NICHD 1999[1][Q]			☒														
**AA or Higher**	Zill 2006[2][K]							■	■									
**Has BA**	Zellman 2008[3][Z]				■													
**BA or Higher**	Early 2006[7][A]								●									
	Henry, Gordon 2003[6][I]											■		■	■			
**Education - Years**	Dunn 1993[8][S]				★						⌘							
	Early 2006[7][A]								★■⌘									
	Epstein 1993[4]						★											

	Hamre 2014[9]	■																
	Montie 2006[10]					■												
	Travers 1980[11]										★							
Education - Ordinal	Clarke-Stewart 1994[12]									⌘								
	Dunn 1993[8][S]				★						⌘							
	Early 2006[7][A]								●									
	Mashburn 2004[13][F]															*O*	*O*	
	Research Triangle 1972[14] – HS 67/68												●					
	Research Triangle 1972[14] – HS 68/69										●		●					●
	Travers 1980[11]										★							

Legend for Table

Significant and Positive	Significant and Negative	Nonsignificant	Statistic
★	★	★	**r - Zero Order Pearson's Correlation**
O	*O*	O	**Beta**
■	■	■	**B (Unstandardized Coefficient)**
❖	❖	❖	**T-Test**
⌘	*⌘*	⌘	**Partial Correlation**
●	●	●	**F-Ratio**
☒	*☒*	☒	**Adjusted Means**

Abbreviations: AA=Associate's Degree; BA=Bachelor's Degree.
[a]This paper is one of a series of Meta-Analyses and Systematic Reviews assessing the relationship between child care quality and children's outcomes; therefore, superscript letters below are in reference to various large databases that samples in these papers were drawn from. These letters have been kept consistent across the series for our readers.
[b]Samples within papers are described in more detail in Table 2 in the manuscript.
[c]Acronyms for child outcomes are listed in Supplemental Information 3.
[d]Identifying Colors (also referred to as Color Naming).
[A]National Center for Early Development and Learning Dataset (NCEDL, 2002, 2004); [F]Georgia Early Childhood Study (GECS, 2002); [I]Georgia Pre-K Program (1996-1997); [K]Head Start Family and Children Experiences Survey (FACES, 2000 Cohort); [Q]National Institute of Child Health and Human Development (NICHD, 1995-1996); [S]8-County Region of North-Central Indiana (Year NR);[T]Otitis Media Study (Year NR); [Z]Colorado QRIS.

Supplemental Information 4
Systematic Review Results: All Outcomes

Table C. Staff Education Predicting all Language Outcomes A to P

**Staff Education Measurement Level**	**STUDY[a, b]**	**LANGUAGE[c]**																	
		ARS - Language/Literacy	COR - Language	DIAL-R - Language	Emergent Literature Composite	Expressive One - Picture Vocabulary	Identifying Letters[d]	Language (author created)	Language (PPVT & WJ Picture Vocabulary)	OWLS - Oral Expression Scale	OWLS - Total Score	PALS-Pre-K - Emergent Literacy	PPVT- Picture Vocabulary	Phoneme Deletion	PLS-3	Pre-CTOPP - Blending Sounds	Pre-CTOPP - Elision	Pre-CTOPP - Print Awareness	Pre-CTOPP - Receptive Vocabulary
**AA or Higher**	Aikens 2010[15][B]												○ ○ ○ ○ ○ ○						
	Aikens 2012[16][M]					↓ ↓ ↓ ↓							↓ ↓ ↓ ↓						
	Zill 2003[17][K]												■						
	Zill 2006[2][K]												■						
**Has a BA**	Aikens 2010[15][B]												○ ○ ○ ○ ○ ○						
	Aikens 2012[16][M]					↓ ↓ ↓ ↓							↓ ↓ ↓ ↓						
	Downer 2012[18] – DLL[A]	■ ■					■■■												

		■																	
	Downer 2012[18] – Latino[A]	■ ■ ■					■■■												
	Early 2007[19] – Head Start[H]												●						
	Early 2007[19] – FACES 2003[L]												●						
	Early 2007[19] – GECS 2002[F]												●						
	Early 2007[19] – MAF 2002-2004[YA, YB]												●						
	Early 2007[19] – NCEDL[A]												●						
	Early 2007[19] – PCER[U]												●						
	Early 2007[19] – NICHD[Q]														●				
	Guo 2014[20][U]												■						
	Henry 2005[21][f]												■						
	Howes 2008[22][A]	■ ★					■★			■★			■ ★						
	Reid 2013[23][A]									○			○						
	Sabol 2013[24][A]						○			○			○						
	Zellman 2008[3][Z]												■						
BA or Higher	Cameron 2011[25]				■ ★														
	Early 2006[7][A]						●			●			●						
	Mashburn, Pianta 2008[26][A]						■			■			■						
	Reid 2013[23][A]									○			○						
	West 2010[27][B]												○ ○						
Education - Years	Burchinal, Nelson 2000[28][D]												■ ■						
	Dotterer 2012[29][A]	■					■				■		■						
	Colwell 2013 [30][N]	○																	
	Early 2006[7][A]						■⌘ ★			■⌘ ★			■ ⌘ ★						

	Epstein 1993[4]		★	★															
	Hamre 2014[9]								■										
	Montie 2006[10]							■											
	Travers 1980[11]												★ ■						
Ordinal	Barnett 2007[31] – Whole Sample						■						■	■					
	Barnett 2007[31] – Spanish						■						■	■					
	Chang 2007[32] – Spanish-Spanish Testing[A]												■						
	Chang 2007[32] – SWEEP (Spanish Children)[A]												■						
	Early 2006[7][A]						●			●			●						
	Early 2007[19] – Head Start[H]												●						
	Early 2007[19] – FACES 2003[L]												●						
	Early 2007[19] – GECS 2002[F]												●						
	Early 2007[19] – NCEDL[A]												●						
	Early 2007[19] – NICHD[Q]														●				
	Early 2007[19] – PCER[U]												●						
	Early 2007[19] – MAF 2002-2004[YA, YB]												●						
	Guo 2014[20][U]												■						
	Lyon 1995[33]												●						
	Mashburn 2010[34][W]											■				■	■	■	■
	Sabol 2013[24][A]						★ ○○			★ ○○			★ ○ ○						
	Son 2013[35][L]												★ ○						
	Travers 1980[11]														■★				

Legend for Table

Significant and Positive	Significant and Negative	Nonsignificant	Statistic
★	★	★	**r - Zero Order Pearson's Correlation**

○	○	○	**Beta**
■	■	■	**B (Unstandardized Coefficient)**
❖	❖	❖	**T-Test**
⌘	⌘	⌘	**Partial Correlation**
●	●	●	**F-Ratio**
⊠	⊠	⊠	**Adjusted Means**
⬇	⬇	⬇	**Effect Size**

Abbreviations: AA=Associate's Degree; BA=Bachelor's Degree.
[a]This paper is one of a series of Meta-Analyses and Systematic Reviews assessing the relationship between child care quality and children's outcomes; therefore, superscript letters below are in reference to various large databases that samples in these papers were drawn from. These letters have been kept consistent across the series for our readers.
[b]Samples within papers are described in more detail in Table 2 in the manuscript.
[c]Acronyms for child outcomes are listed in Supplemental Information 3.
[d]Identifying Letters (also referred to as Alphabet Recognition Test; Letter-Naming Test, Naming Letters).
[A]National Center for Early Development and Learning Dataset (NCEDL, 2002, 2004); [B]Head Start Family and Children Experiences Survey (FACES, 2006 Cohort); [D]Cost, Quality and Outcomes Study (CQO, 1993-1994); [F]Georgia Early Childhood Study (GECS, 2002); [H]Early Head Start (EHS, 2001-2003 Cohort); [K]Head Start Family and Children Experiences Survey (FACES, 2000 Cohort); [L]Head Start Family and Children Experiences Survey (FACES, 2003 Cohort); [M]Head Start Family and Children Experiences Survey (FACES, 2009 Cohort); [N]Early Childhood Longitudinal Study (ECLS-B, 2001-2006 Birth Cohort); [Q]National Institute of Child Health and Human Development (NICHD, 1995-1996); [U]Preschool Curriculum Evaluation Research (PCER, 1999-2003); [W]Mid Atlantic State US (Year 2004-2005); [YA]More is Four North Carolina Study (2002-2003 Cohort); [YB]More is Four North Carolina Study (2003-2004 Cohort); [Z]Colorado QRIS.

Supplemental Information 4
Systematic Review Results: All Outcomes

Table D. Staff Education Prediction all Language Outcomes P to W

Staff Education Measurement Level	STUDY[a, b]	LANGUAGE[c]																
		Pre-LAS 2000	RDLS - Expressive Language	RDLS - Language Comprehension	Rhyme Recognition	SICD-Expressive Communication Age	SICD-Receptive Communication Age	SRF - Communication Skills	Stanford 9 - Language Arts	Story & Print Concepts – Book Knowledge	Story & Print Concepts – Total Score	TOPEL (PA + PK)	WJ - Academic Knowledge	WJ - Dictation	WJ - Letter Word ID	WJ - Passage Comprehension	WJ - Picture Vocabulary	WJ - Rhyming
College or Higher	Burchinal, Roberts 2000[5][T]					★	★											
	Henry, Gordon 2003[6][I]								■									
	NICHD 1999[1][Q]		☒	☒														
AA or Higher	Aikens 2010[15][B]														○○○○○○			
	Aikens 2012[16][M]														↓↓↓↓			
	Zill 2003[17][K]													■	■			
	Zill 2006[2][K]									■				■	■			
Has a BA	Aikens 2010[15][B]														○○○○○○			
	Aikens 2012[15][M]														↓↓↓↓			
	Early 2007[19] – Head Start[H]														●			
	Early 2007[19] – FACES 2003[L]														●			
	Early 2007[19] – GECS 2002[F]														●			
	Early 2007[19] – NCEDL[A]														●			
	Early 2007[19] – NICHD[Q]														●			
	Early 2007[19] – PCER[U]														●			
	Henry 2005[21][F]										■							
	Sabol 2013[24][A]																	○
	Zellman 2008[3][Z]														■	■		
BA or Higher	Cameron 2011[25]							`					■★				■★	
	Early 2006[7][A]																	●
	Henry, Gordon 2003[6][I]								■									
	Mashburn, Pianta 2008[26][A]																	■
	West 2010[27][B]														○○			

Education - Years	Early 2006[7][A]																	■⌘★
	Dotterer 2012[29][A]																	■
	Hamre 2014[9]										■							
Ordinal	Barnett 2007[31] - Whole Sample				■												■	
	Barnett 2007[31] - Spanish				■												■	
	Chang 2007[32][A]	■																
	Early 2006[7][A]																	●
	Early 2007[19] – Head Start[H]														●			
	Early 2007[19] – FACES 2003[L]														●			
	Early 2007[19] – GECS 2002[F]														●			
	Early 2007[19] – NCEDL[A]														●			
	Early 2007[19] – NICHD[Q]														●			
	Early 2007[19] – PCER[U]														●			
	Hindman 2010[36][J]													■				
	Mashburn 2004[13][F]							*o*										
	Sabol 2013[24][A]																	★ oo
	Son 2013[35][L]														o★			

Legend for Table

Significant and Positive	Significant and Negative	Nonsignificant	Statistic
★	*★*	★	r - Zero Order Pearson's Correlation
o	*o*	o	Beta
■	*■*	■	B (Unstandardized Coefficient)
⌘	*⌘*	⌘	Partial Correlation
●	*●*	●	F-Ratio
⊠	*⊠*	⊠	Adjusted Means
🡻	*🡻*	🡻	Effect Size

Abbreviations: AA=Associate's Degree; BA=Bachelor's Degree.

[a]This paper is one of a series of Meta-Analyses and Systematic Reviews assessing the relationship between child care quality and children's outcomes; therefore, superscript letters below are in reference to various large databases that samples in these papers were drawn from. These letters have been kept consistent across the series for our readers.

[b]Samples within papers are described in more detail in Table 2 in the manuscript.

[c]Acronyms for child outcomes are listed in Supplemental Information 3.

[A]National Center for Early Development and Learning Dataset (NCEDL, 2002, 2004); [B]Head Start Family and Children Experiences Survey (FACES, 2006 Cohort); [F]Georgia Early Childhood Study (GECS, 2002); [H]Early Head Start (EHS, 2001-2003 Cohort); [I]Georgia Pre-K Program (1996-1997); [J]Head Start Family and Children Experiences Survey (FACES, 1997 Cohort); [K]Head Start Family and Children Experiences Survey (FACES, 2000 Cohort); [L]Head Start Family and Children Experiences Survey (FACES, 2003 Cohort); [M]Head Start Family and Children Experiences Survey (FACES, 2009 Cohort); [Q]National Institute of Child Health and Human Development (NICHD, 1995-1996); [T]Otitis Media Study (Year NR); [U]Preschool Curriculum Evaluation Research (PCER, 1999-2003); [Z]Colorado QRIS.

Supplemental Information 4
Systematic Review Results: All Outcomes

Table E. Staff Education Prediction all Math Outcomes

**Staff Education Measurement Level**	**STUDY**[a, b]	**Math Outcomes**[c]						
		COR - Logic & Math	ECLS-B Math	Identifying Numbers[d]	One-One Counting	Stanford 9 - Math	TEMA -3	WJ - Applied Problems
**College or Higher**	Henry, Gordon 2003[I]					■		
**AA or Higher**	Aikens 2010[15][B]		○○○○○○					○○○○○○
	Aikens 2012[16][M]							↓↓↓↓
	Zill 2006[2][K]				■			■
**Has a BA**	Aikens 2010[15][B]		○○○○○○					○○○○○○
	Aikens 2012[16][M]							↓↓↓↓
	Choi 2014[37]						○	
	Downer 2012[18] – DLL[A]							■■■
	Downer 2012[18] – Latino[A]							■■■
	Early 2007[19] – Head Start[H]							●
	Early 2007[19] – FACES 2003[L]							●
	Early 2007[19] – GECS 2002[F]							●
	Early 2007[19] – MAF 2002-2004[YA, YB]							●
	Early 2007[19] – NCEDL[A]							●
	Early 2007[19] – NICHD[Q]							●
	Early 2007[19] – PCER[U]							●
	Henry 2005[21][F]							■
	Howes 2008[22][A]							■★

	Reid 2013[23][A]							○
	Sabol 2013[24][A]							○
	Zellman 2008[3][Z]							□
BA or Higher	Cameron 2011[25]							□☆
	Early 2006[7][A]			•				↓
	Henry 2003[6][I]					□		
	Kim 2011[38][L]							★
	Mashburn, Pianta 2008[26][A]							□
	Reid 2013[23][A]							○
	West 2010[27][B]		○					○○
Education - Years	Colwell 2013[30][N]		○					
	Dotterer 2012[29][A]			■				□
	Early 2006[7][A]			★□⌘				★■⌘
	Epstein 1993[4]	☆						
Education - Ordinal	Barnett 2007[31] - Whole Sample							□
	Barnett 2007[31] - Spanish							□
	Early 2006[7][A]			•				●
	Early 2007[19] - Head Start[H]							•
	Early 2007[19] - FACES 2003[L]							•
	Early 2007[19] - GECS 2002[F]							•
	Early 2007[19] - MAF 2002-2004[YA, YB]							•
	Early 2007[19] - NCEDL[A]							●
	Early 2007[19] - NICHD[Q]							•
	Early 2007[19] - PCER[U]							•
	Hindman 2010[36][J]							□
	Sabol 2013[24][A]							☆○○
	Son 2013[35][L]							☆○

Legend for Table

Significant & Positive	Significant & Negative	Nonsignificant	Statistic
★	★	☆	**r - Zero Order Pearson's Correlation**
⭘	⬯	○	**Beta**
■	▰	□	**B (Unstandardized Coefficient)**

⌘	⌘	⌘	**Partial Correlation**
●	●	●	**F-Ratio**
➘	➘	➘	**Effect Size**

Abbreviations: AA=Associate's Degree; BA=Bachelor's Degree.

[a]This paper is one of a series of Meta-Analyses and Systematic Reviews assessing the relationship between child care quality and children's outcomes; therefore, superscript letters below are in reference to various large databases that samples in these papers were drawn from. These letters have been kept consistent across the series for our readers.

[b]Samples within papers are described in more detail in Table 2 in the manuscript.

[c]Acronyms for child outcomes are listed in Supplemental Information 3.

[d]Identifying Numbers (also referred to as Naming Numbers).

[A]National Center for Early Development and Learning Dataset (NCEDL, 2002, 2004); [B]Head Start Family and Children Experiences Survey (FACES, 2006 Cohort); [F]Georgia Early Childhood Study (GECS, 2002); [H]Early Head Start (EHS, 2001-2003 Cohort); [I]Georgia Pre-K Program (1996-1997); [J]Head Start Family and Children Experiences Survey (FACES, 1997 Cohort); [K]Head Start Family and Children Experiences Survey (FACES, 2000 Cohort); [L]Head Start Family and Children Experiences Survey (FACES, 2003); [M]Head Start Family and Children Experiences Survey (FACES, 2009 Cohort); [N]Early Childhood Longitudinal Study (ECLS-B, 2001-2006 Birth Cohort); [Q]National Institute of Child Health and Human Development (NICHD, 1995-1996); [U]Preschool Curriculum Evaluation Research (PCER, 1999-2003); [YA]More is Four North Carolina Study (2002-2003 Cohort); [YB]More is Four North Carolina Study (2003-2004 Cohort); [Z]Colorado QRIS.

Supplemental Information 4
Systematic Review Results: All Outcomes

Table F. Staff Education Predicting all Social-Emotional Positive Behavior Outcomes A to H

Staff Education Measurement Level	STUDY[a, b]	SOCIAL-EMOTIONAL POSITIVE BEHAVIOR[c]												
		CBI - Considerateness	CBI - Sociability	Comply with Parents	Comply with Requests	Cooperative Behavior	COR - Social Relations	ECLS-B - Attention & Concentration	ECLS-B - Emotional & Behavioral Regulation	ECLS-B - Prosocial	ECLS-K Approaches to Learning	General Compliance	Gumpgookies	Head-to-Toes Task
AA or Higher	Aikens 2012[16][M]										⬇⬇⬇			
	Zill 2003[17][K]					□								
Has a BA	Aikens 2012[16][M]										⬇⬇⬇			
	Zellman 2008[3][Z]	□												
BA or Higher	Cameron 2011[25]													☆□
Education - Years	Colwell 2013[30][N]							**O**○	**O**○	○○				
	Dunn 1993[8][S]		☆											
	Epstein 1993[4]						☆							
Education - Ordinal	Clarke-Stewart 1994[12]			⌘	⌘							⌘		
	Dunn 1993[8][S]		☆											
	Research Triangle 1972[14] – HS 68/69												●	

Legend for Table

Significant and Positive	Significant and Negative	Nonsignificant	Statistic
★	*★*	☆	**r - Zero Order Pearson's Correlation**
**O**	*O*	○	**Beta**
■	*■*	□	**B (Unstandardized Coefficient)**

⌘	⌘	⌘	**Partial Correlation**
●	●	●	**F-Ratio**
🡻	🡻	🡻	**Effect Size**

Abbreviations: AA=Associates Degree; BA=Bachelor's Degree.

[a]This paper is one of a series of Meta-Analyses and Systematic Reviews assessing the relationship between child care quality and children's outcomes; therefore, superscript letters below are in reference to various large databases that samples in these papers were drawn from. These letters have been kept consistent across the series for our readers.

[b]Samples within papers are described in more detail in Table 2 in the manuscript.

[c]Acronyms for child outcomes are listed in Supplemental Information 3.

[K]Head Start Family and Children Experiences Survey (FACES, 2000 Cohort); [M]Head Start Family and Children Experiences Survey (FACES, 2009 Cohort); [N]Early Childhood Longitudinal Study (ECLS-B, 2001-2006 Birth Cohort); [S]8-County Region of North-Central Indiana (Year NR); [Z]Colorado QRIS.

Supplemental Information 4
Systematic Review Results: All Outcomes

Table G. Staff Education Predicting all Social-Emotional Positive Behavior Outcomes P to Z

Staff Education Measurement Level	STUDY[a, b]	SOCIAL-EMOTIONAL POSITIVE BEHAVIOR[c]											
		Pencil Tap Task	PLBS – Learning Behavior	Positive Behavior (author created)	Social Cognitive Ability	Social Competence with Stranger	Social Competence with Visitor	Social Skills (author created)	Social Skills & Positive Approach to Learning	SSPBS -Social Competence	SSRS - Social Skills	STRS - Closeness	TCRS - Social Competence
College or Higher	NICHD 1999[1][Q]			☒									
AA or Higher	Aikens 2010[15][B]										○○○○○○		
	Aikens 2012[16][M]	↓↓↓									↓↓↓		
	Zill 2006[2][B]										■■		
Has a BA	Aikens 2010[15][B]										○○○○○○		
	Aikens 2012[16][M]	↓↓↓									↓↓↓		
	Downer 2012[18] – DLL[A]												■■■
	Downer 2012[18] - Latino[A]												■■■
	Howes 2008[22][A]									■★			
	Reid 2013[23][A]												○
	Sabol 2013[24][A]												○
BA or Higher	Mashburn 2008[39][A]												■
	Reid 2013[23][A]												○
	West 2010[27][B]										○		

**Education - Years**	Hamre 2014[9]	■										■	
**Education - Ordinal**	Clarke-Stewart 1994[12]				⌘	⌘	⌘						
	Sabol 2013[24][A]												○○★
	Son 2013[35][L]		○★					○★	○★				

Legend for Table

**Significant and Positive**	**Significant and Negative**	**Nonsignificant**	**Statistic**
★	★	★	**r - Zero Order Pearson's Correlation**
○	○	○	**Beta**
■	■	■	**B (Unstandardized Coefficient)**
⌘	⌘	⌘	**Partial Correlation**
☒	☒	☒	**Adjusted Means**
🡻	🡻	🡻	**Effect Size**

Abbreviations: AA=Associates Degree; BA=Bachelor's Degree.

[a]This paper is one of a series of Meta-Analyses and Systematic Reviews assessing the relationship between child care quality and children's outcomes; therefore, superscript letters below are in reference to various large databases that samples in these papers were drawn from. These letters have been kept consistent across the series for our readers.

[b]Samples within papers are described in more detail in Table 2 in the manuscript.

[c]Acronyms for child outcomes are listed in Supplemental Information 3.

[A]National Center for Early Development and Learning Dataset (NCEDL, 2002, 2004); [B]Head Start Family and Children Experiences Survey (FACES, 2006 Cohort); [L]Head Start Family and Children Experiences Survey (FACES, 2003) Cohort; [M]Head Start Family and Children Experiences Survey (FACES, 2009) Cohort; [Q]National Institute of Child Health and Human Development (NICHD, 1995-1996).

Supplemental Information 4

Table H. Staff Education Predicting all Social-Emotional Problem Behavior Outcomes

Staff Education Measurement Level	STUDY[a,b]	SOCIAL-EMOTIONAL PROBLEM BEHAVIOR[c]															
		Behavior Problem	Behavior Problems (author created)	Caregiver Staff Report Form/25	CBI - Apathy	CBI - Hostility	FACES - Aggressive	FACES - Hyperactive	FACES - Withdrawn	Feeling of Inadequacy	Motivational Problem	PBQ - Hyperactivity-Distractible	PBS - Hyperactive Behavior	SSPBS - Behavior Problems	SSRS - Problem Behaviors	STRS - Conflict	TCRS - Behavior Problems
College or Higher	NICHD 1999[1][Q]		⊠														
AA or Higher	Aikens 2010[15][B]		○○○○○○														
	Aikens 2012[16][M]		↓↓↓														
	Zill 2003[17][K]												■				
	Zill 2006[2][K]						■■	■⬛	■⬛								
Has a BA	Aikens 2010[15][B]		○○○○○○														
	Aikens 2012[16][M]		↓↓↓														
	Downer 2012[18] – DLL[A]																■■■
	Downer 2012[18] – Latino[A]																■■■
	Howes 2008[22][A]													■★			
	Sabol 2013[24][A]																○
	Zellman				■	■											

	2008[3][Z]																
BA or Higher	Mashburn 2008[39][A]																■
	West 2010[27][B]														○		
Education - Years	Dunn 1993[8][S]											★					
	Hamre 2014[9]															■	
Education - Ordinal	Dunn 1993[8][S]											★					
	Kaiser 2002[40]			■													
	Research 1972[14] – HS 67/68	●								●	●						
	Research Triangle 1972[14] – HS 68/69	●								●	●						
	Sabol 2013[24][A]																○○★

Legend for Table

Significant and Positive	Significant and Negative	Nonsignificant	Statistic
★	★	★	**r - Zero Order Pearson's Correlation**
○	○	○	**Beta**
■	■	■	**B (Unstandardized Coefficient)**
⌘	⌘	⌘	**Partial Correlation**
●	●	●	**F-Ratio**
⊠	⊠	⊠	**Adjusted Means**
⬇	⬇	⬇	**Effect Size**

Abbreviations: AA=Associates Degree; BA=Bachelor's Degree.
[a]This paper is one of a series of Meta-Analyses and Systematic Reviews assessing the relationship between child care quality and children's outcomes; therefore, superscript letters below are in reference to various large databases that samples in these papers were drawn from. These letters have been kept consistent across the series for our readers.
[b]Samples within papers are described in more detail in Table 2 in the manuscript.
[c]Acronyms for child outcomes are listed in Supplemental Information 3.
[A]National Center for Early Development and Learning Dataset (NCEDL, 2002, 2004); [B]Head Start Family and Children Experiences Survey (FACES, 2006 Cohort); [K]Head Start Family and Children Experiences Survey (FACES, 2000 Cohort); [M]Head Start Family and Children Experiences Survey (FACES, 2009 Cohort); [Q]National Institute of Child Health and Human Development (NICHD, 1995-1996); [S]8-County Region of North-Central Indiana (Year NR); [Z]Colorado QRIS.

References

1. NICHD Early Child Care Research Network. Child outcomes when child care center classes meet recommended standards for quality. Am J Public Health. 1999;89(7): 1072-1077.

2. Zill N, Resnick G, Kim K, O'Donnell K, Sorongon A, Ziv Y, et al. Head Start Performance Measures Center Family and Child Experiences Survey (FACES 2000): Technical Report. Washington, DC: Office of Planning, Research and Evaluation, Administration for Children and Families, U.S. Department of Health and Human Services; 2006. Available: http://www.acf.hhs.gov/sites/default/files/opre/tech2k_final2.pdf.

3. Zellman G, Perlman M, Le V, Setodji C. Assessing the Validity of the Qualistar Early Learning Quality Rating and Improvement System as a Tool for Improving Child-Care Quality. Santa Monica, CA: RAND Corporation; 2008. Available: http://www.rand.org/content/dam/rand/pubs/monographs/2008/RAND_MG650.pdf. Accessed July 1, 2015.

4. Epstein A. Training for Quality Improving Early Childhood Programs through Systematic Inservice Training. Ypsilanti, Michigan: High/ Scope Press; 1993.

5. Burchinal M, Roberts J, Riggins Jr R, Zeisel S, Neebe E, Bryant D. Relating quality of center-based child care to early cognitive and language development longitudinally. Child Dev. 2000;71(2): 339-357. doi:10.1111/1467-8624.00149.

6. Henry G, Gordon C, Henderson L, Ponder B. *G*eorgia Pre-K Longitudinal Study: Final Report, 1996-2001. Atlanta, GA: Andrew Young School of Policy Studies, Georgia State University; 2003. Available: http://files.eric.ed.gov/fulltext/ED481260.pdf.

7. Early D, Bryant D, Pianta R, Clifford R, Burchinal M, Ritchie S, et al. Are Staffs' education, major, and credentials related to classroom quality and children's academic gains in pre-kindergarten? Early Child Res Q. 2006;21(2): 174-195. doi:10.1016/j.ecresq.2006.04.004.

8. Dunn L. Proximal and distal features of day care quality and children's development. Early Child Res Q. 1993;8(2): 167-192. doi:10.1016/S0885-2006(05)80089-4.

9. Hamre B, Hatfield B, Pianta R, Jamil F. Evidence for general and domain-specific elements of Staff–child interactions: associations with preschool children's development. Child Dev. 2014;85(3): 1257-1274. doi:10.1111/cdev.12184.

10. Montie J, Xiang Z, Schweinhart L. Preschool experience in 10 countries: cognitive and language performance at age 7. Early Child Res Q. 2006;21(3): 313-331. doi:10.1016/j.ecresq.2006.07.007.

11. Travers J, Goodson B, Singer J, Connell D. Research Results of the National Day Care Study. Final Report of the National Day Care Study. Volume II. Cambridge, MA: Abt Associates, Inc.; 1980. http://eric.ed.gov/?id=ED195336. Accessed July 1, 2015.

12. Clarke-Stewart K, Gruber C, Fitzgerald L. Children at Home and in Day Care. Hillsdale, NJ: Lawrence Erlbaum Associates, Inc; 1994.

13. Mashburn A, Henry G. Assessing school readiness: validity and bias in preschool and kindergarten Staffs' ratings. Educ Meas Issues Pract. 2004;23(4):16-30. doi:10.1111/j.1745-3992.2004.tb00165.x.

14. Research Triangle Institute. A Report on Two National Samples of Head Start Classes: Some Aspects of Child Development Participants in Full Year 1967-68 and 1968-69 Programs. Durham, NC: Research Triangle Institute; 1972. Available: http://files.eric.ed.gov/fulltext/ED085407.pdf.

15. Aikens N, Tarullo L, Husley L, Ross C, West J, Xue Y. A Year in Head Start: Children, Families and Programs. Washington, DC: U.S. Department of Health and Human Services, Administration for Children and Families, Office of Planning, Research and Evaluation; 2010. Available: http://www.acf.hhs.gov/sites/default/files/opre/year_final.pdf. Accessed July 1, 2015.

16. Aikens N, Moiduddin E, Xue Y, Tarullo L, West J. Data Tables for Child Outcomes and Classroom Quality in FACES 2009 Report. Washington, DC: U.S. Department of Health and Human Services, Administration for Children and Families, Office of Planning, Research and Evaluation; 2012. Available: http://www.acf.hhs.gov/sites/default/files/opre/data_tables_for_child_outcomes_and_classroom_quality_in_faces_2009.pdf. Accessed July 1, 2015.

17. Zill N, Resnick G, Kim K, O'Donnell K, Sorongon A, McKey R, et al. Head Start FACES 2000: A Whole-Child Perspective on Program Performance. Washington, DC: Administration for Children and Families, U.S. Department of Health and Human Services; 2003. Available: http://www.acf.hhs.gov/sites/default/files/opre/faces00_4thprogress.pdf. Accessed July 1, 2015.

18. Downer J, López M, Grimm K, Hamagami A, Pianta R, Howes C. Observations of Staff-child interactions in classrooms serving Latinos and dual language learners: applicability of the classroom assessment scoring system in diverse settings. Early Child Res Q. 2012;27(1): 21-32. doi:10.1016/j.ecresq.2011.07.005.

19. Early D, Maxwell K, Burchinal M, Alva S, Bender R, Bryant D, et al. Staffs' education, classroom quality, and young children's academic skills: results from seven studies of preschool programs. Child Dev. 2007;78(2): 558-580. doi:10.1111/j.1467-8624.2007.01014.x.

20. Guo Y, Tompkins V, Justice L, Petscher Y. Classroom age composition and vocabulary development among at-risk preschoolers. Early Educ Dev. 2014;25(7): 1016-1034. doi:10.1080/10409289.2014.893759.

21. Henry G, Rickman D, Ponder B, Henderson L, Mashburn A, Gordon C. The Georgia Early Childhood Study, 2001-2004, Final Report. Atlanta, GA: Andrew Young School of Policy Studies, Georgia State University; 2005. Available: http://citeseerx.ist.psu.edu/viewdoc/download?doi=10.1.1.177.2555&rep=rep1&type=pdf.

22. Howes C, Burchinal M, Pianta R, Bryant D, Early D, Clifford R, et al. Ready to learn? children's pre-academic achievement in pre-kindergarten programs. Early Child Res Q. 2008;23(1):27-50. doi:10.1016/j.ecresq.2007.05.002.

23. Reid J, Ready D. High-quality preschool: the socioeconomic composition of preschool classrooms and children's learning. Early Educ Dev. 2013;24(8): 1082-1111.

24. Sabol T, Hong S, Pianta R, Burchinal M. Can rating pre-k programs predict children's learning? Science. 2013;341(6148): 845-846. doi:10.1126/science.1233517.

25. Cameron C, Morrison F. Staff activity orienting predicts preschoolers academic and self regulatory skills. Early Educ Dev. 2011;22(4): 620-648. doi:10.1080/10409280903544405.

26. Mashburn AJ, Pianta RC, Hamre BK, Downer J, Barbarin O, Bryant D, et al. Measures of classroom quality in prekindergarten and children's development of academic, language, and social skills. Child Dev. 2008;79(3): 732-749. doi:10.1111/j.1467-8624.2008.01154.x.

27. West J, Malone L, Hulsey L, Aikens N, Tarullo L. Head Start Children Go to Kindergarten. Washington, DC: U.S. Department of Health and Human Services, Administration for Children and Families, Office of Planning, Research and Evaluation; 2010. Available: http://www.acf.hhs.gov/sites/default/files/opre/hs_kindergarten.pdf. Accessed July 1, 2015.

28. Burchinal M, Nelson L. Family selection and child care experiences: implications for studies of child outcomes. Early Child Res Q. 2000;15(3): 385-411. doi:10.1016/S0885-2006(00)00072-7.

29. Dotterer A, Burchinal M, Bryant D, Early D, Pianta R. Universal and targeted pre-kindergarten programmes: a comparison of classroom characteristics and child outcomes. Early Child Dev Care. 2013;183(7): 931-950. doi:10.1080/03004430.2012.698388.

30. Colwell N, Gordon R, Fujimoto K, Kaestner R, Korenman S. New evidence on the validity of the Arnett Caregiver Interaction Scale: results from the early childhood longitudinal study-birth cohort. Early Child Res Q. 2013;28(2): 218-233. doi:10.1016/j.ecresq.2012.12.004.

31. Barnett W, Yarosz D, Thomas J, Jung K, Blanco D. Two-way and monolingual english immersion in preschool education: an experimental comparison. Early Child Res *Q*. 2007;22(3): 277-293. doi:10.1016/j.ecresq.2007.03.003.

32. Chang F, Crawford G, Early D, Bryant D, Howes C, Burchinal M, et al. Spanish-speaking children's social and language development in pre-kindergarten classrooms. Early Educ Dev. 2007;18(2): 243-269. doi:10.1080/10409280701282959.

33. Lyon M, Canning P. Atlantic Day Care Study. Halifax: Mount Saint Vincent University; 1995.

34. Mashburn A, Downer J, Hamre B, Justice L, Pianta R. Consultation for Staffs and children's language and literacy development during pre-kindergarten. Appl Dev Sci. 2010;14(4): 179-196. doi:10.1080/10888691.2010.516187.

35. Son S, Kwon K, Jeon H, Hon S. Head Start classrooms and children's school readiness benefit from Staffs' qualifications and ongoing training. Child Youth Care Forum. 2013;42(6): 525-553. doi:10.1007/s10566-013-9213-2.

36. Hindman A, Skibbe L, Miller A, Zimmerman M. Ecological contexts and early learning: contributions of child, family, and classroom factors during head start, to literacy and mathematics growth through first grade. Early Child Res Q. 2010;25(2): 235-250. doi:10.1016/j.ecresq.2009.11.003.

37. Choi J, Dobbs-Oates J. Childcare quality and preschoolers' math development. Early Child Dev Care. 2014;184(6): 915-932. doi:10.1080/03004430.2013.829822.

38. Kim S, Chang M, Kim H. Does Staff educational training help the early math skills of English language learners in Head Start? Child Youth Serv Rev. 2011;33(5): 732-740. doi:10.1016/j.childyouth.2010.11.019.

39. Mashburn A. Quality of social and physical environments in preschools and children's development of academic, language and literacy skills. Appl Dev Sci. 2008;12(3): 103-127. doi:10.1080/10888690802199392.

40. Kaiser A, Cai X, Hancock T, Foster E. Staff-reported behavior problems and language delays in boys and girls enrolled in head start. Counc Child Behav Disord. 2002;28(1): 23-39.
