# Supplementary material for: Education of staff in preschool aged classrooms in child care centers and child outcomes: A meta-analysis and systematic review
Source: PLoS One. 2017 Aug 30;12(8):e0183673. doi: 10.1371/journal.pone.0183673 (PMC5576714; doi:10.1371/journal.pone.0183673)
Supplement: S5 File — (PDF) [file pone.0183673.s005.pdf]

Education of Staff in Preschool Aged Classrooms in Child Care Centers and Child Outcomes: A Meta-Analysis and Systematic Review

Supplemental Information 5

List of Acronyms Used in the Description of Studies Table	
**Acronym Category**	**Full Name**
**Education**	Associate's Degree (AA)<br>Bachelor's Degree (BA)<br>Child Development Associate (CDA)<br>Master's Degree (MA)
**Journals**	American Journal of Public Health (AJPH)<br>Applied Developmental Psychology (ADP)<br>Behavioral Development (BD)<br>Child and Youth Care Forum (CYCF)<br>Child Development (CD)<br>Children and Youth Services Review (CYSR)<br>Early Childhood Development (ECD)<br>Early Childhood Development and Care (ECDC)<br>Early Childhood Research Quarterly (ECRQ)<br>Early Education and Development (EED)<br>Education Measurement: Issues and Practice (EMIP)
**Large Samples**	Cost Quality Outcome Study (CQO)<br>Early Childhood Longitudinal Study (ECLS)<br>Early Childhood Study – Birth Cohort (ECLS-B)<br>Early Childhood Study – Kindergarten Cohort (ECLS-K)<br>Early Head Start (EHS)<br>Effective Provision of Pre-School Education (EPPE)<br>Family and Child Experiences Survey (FACES)<br>Georgia Early Childhood Study (GECS)<br>Georgia More at Four Study (MAF)<br>Head Start (HS)<br>Head Start Family and Children Experiences Study (FACES)<br>National Center for Early Development and Learning (NCEDL)<br>National Day Care Study (NDCS)<br>National Institute of Child Health and Human Development Study of Early Child Care and Youth Development (NICHD SECCYD)<br>Otitis Media Study (OMS)<br>Preschool Curriculum Evaluation Research (PCER)<br>State-Wide Early Education Program Study (SWEEP)
**Covariates & Other**	Caregiver Interaction Scale (CIS)<br>CLASS (Classroom Assessment Scoring System)<br>Developmentally Appropriate Beliefs (DAP)<br>Dual Language Learner (DLL)<br>Early Childhood Education (ECE)<br>Early Childhood Education and Care (ECEC)

	Early Childhood Experiences Rating Scale (ECERS_R)<br>English as a Second Language Status (ESL)<br>English Language Learner (ELL)<br>Family Child care Environment Rating Scale (FCCERS)<br>Individual Education Plan (IEP)<br>International Association for the Evaluation of Educational Achievement (IEA)<br>Not Reported (NR)<br>Quality Ratings and Improvement Systems (QRISs)<br>Socio-Economic Status (SES)<br>Temporary Assistance for Needy Families (TANF)<br>Woman, Infants, and Children (WIC) Food and Nutrition Service

Note: See also S3 File Child Outcomes.
